# Unraveling the Major Differences between the Trinuclear Cyclopentadienylmetal Carbonyl Chemistry of Cobalt and That of Nickel—A Theoretical Study

**DOI:** 10.1021/acsomega.3c02849

**Published:** 2023-07-06

**Authors:** Yuexia Lin, Hongyan Wang, R. Bruce King

**Affiliations:** † School of Physical Science and Technology, Key Laboratory of Advanced Technologies of Materials, Ministry of Education of China, Southwest Jiaotong University, Chengdu 610031, China; ‡ Department of Chemistry and Center for Computational Chemistry, University of Georgia, Athens, Georgia 30602, United States

## Abstract

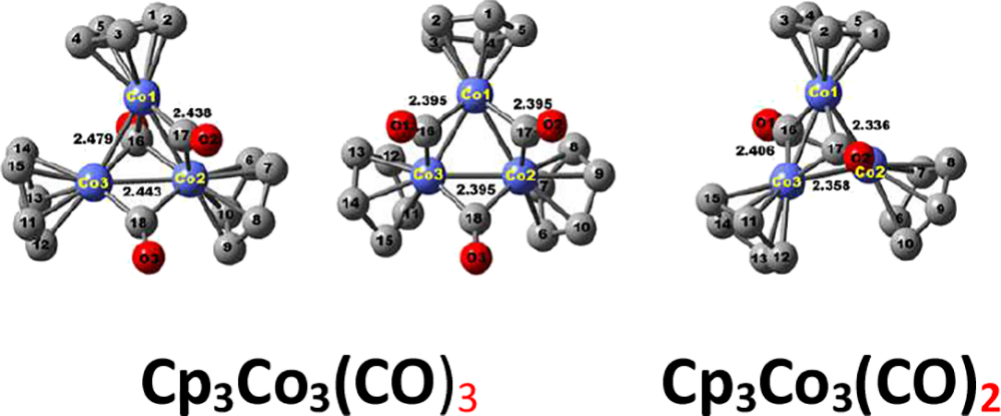

The geometries and energetics of the trinuclear cyclopentadienylmetal carbonyls Cp_3_M_3_(CO)_*n*_ (Cp = η^5^-C_5_H_5_); M = Co, Ni; *n* = 3, 2, 1, 0) have been investigated by density functional theory. The cobalt and nickel systems are found to be rather different owing to the different electronic configurations of the metal atoms. For cobalt, the small calculated energy separation of 5.0 kcal/mol between the two lowest-energy singlet Cp_3_Co_3_(μ_3_-CO)(μ-CO)_2_ and Cp_3_Co_3_(μ-CO)_3_ tricarbonyl structures accounts for the experimental results of both isomers as stable species that can be isolated and structurally characterized by X-ray crystallography. The corresponding Cp_3_Ni_3_(CO)_3_ species in the nickel system are predicted not to be viable owing to exothermic CO dissociation to give the experimentally observed very stable Cp_3_Ni_3_(μ-CO)_2_, which is found to be the lowest-energy isomer by a substantial margin of ∼25 kcal/mol. In all of the low-energy Cp_3_M_3_(CO)_*n*_ (*n* = 2, 1) structures, including that of the experimentally known triplet spin state Cp_3_Co_3_(μ_3_-CO)_2_, all of the carbonyl groups are face-bridging or face-semi-bridging μ_3_-CO groups bonded to all three metal atoms of the M_3_ triangle. In the lowest-energy carbonyl-free Cp_3_M_3_ (M = Co, Ni) structures, agostic C–H–M interactions are found using hydrogens of the Cp rings. In addition, the lowest-energy Cp_3_Ni_3_ is the only structure among all of the low-energy Cp_3_M_3_(CO)_*n*_ (M = Co, Ni; *n* = 3, 2, 1, 0) structures in which each Cp ring is a bridging rather than terminal ligand.

## 1 Introduction

The chemistry of binary metal carbonyls dates back to the 1890 discovery of Ni(CO)_4_ by Mond et al.^1^ Shortly thereafter, the first binary iron carbonyl Fe(CO)_5_ and its photolysis product Fe_2_(CO)_9_ were discovered by the same research group.^2^ A third iron carbonyl of stoichiometry [Fe(CO)_4_]_*n*_ was first synthesized by Dewar and Jones^3^ and shown to be the trimeric Fe_3_(CO)_12_ by Hieber and Becker^4^ in 1930 using cryoscopy in Fe(CO)_5_. The trimeric nature of Fe_3_(CO)_12_, showing a central Fe_3_ triangle with ten terminal CO groups and two bridging CO groups, was confirmed by X-ray diffraction in 1966 by Wei and Dahl after considerable disorder problems.^5,6^ More accurate geometrical parameters for Fe_3_(CO)_12_ were determined by Cotton and Troup in 1974 using improved X-ray crystallographic methods.^7^

The seminal discovery of ferrocene, Cp_2_Fe (Cp = η^5^-C_5_H_5_), in 1951^8,9^ was soon followed by the discovery of a series of cyclopentadienyl metal carbonyl derivatives. The pentahapto coordination of the Cp ring to the metal atom in such species involves a donation of three electron pairs from the cyclopentadienide anion to the metal atom through one σ-type interaction and two orthogonal π-type interactions. For example, the anions CpM(CO)_3_^–^ (M = Cr, Mo, W)^10,11^ are analogues of the corresponding metal hexacarbonyls M(CO)_6_. Similarly, the neutral mononuclear cyclopentadienylmetal carbonyls CpMn(CO)_3_ and CpCo(CO)_2_ can be considered as analogues of Cr(CO)_6_ and Fe(CO)_5_, respectively. In such comparisons of neutral cyclopentadienyl metal carbonyl derivatives with neutral binary metal carbonyl derivatives, the central metal in the cyclopentadienylmetal carbonyl lies one position to the right in the Periodic Table relative to the central metal atom in the corresponding binary metal carbonyl.

The three cyclopentadienylcobalt carbonyl derivatives analogous to the three known binary iron carbonyls Fe(CO)_5_, Fe_2_(CO)_9_, and Fe_3_(CO)_12_ are CpCo(CO)_2_, Cp_2_Co_2_(CO)_3_, and Cp_3_Co_3_(CO)_3_, respectively. A second binuclear cyclopentadienylcobalt carbonyl, namely, Cp_2_Co_2_(CO)_2_ with a formal Co=Co double bond,^12^ is also known analogous to an iron carbonyl, Fe_2_(CO)_8_. A second rather unstable triplet state Cp_3_Co_3_(CO)_2_ is known that would be the analogue of the unknown Fe_3_(CO)_11_.^13^ Finally, a tetranuclear cyclopentadienylcobalt carbonyl, Cp_4_Co_4_(CO)_2_, is known analogous to an iron carbonyl Fe_4_(CO)_14_. However, neither Fe_2_(CO)_8_ nor Fe_4_(CO)_14_ has been synthesized as stable species under ambient conditions.^14^ They have only been observed in low-temperature matrices.

The mononuclear cyclopentadienylcobalt dicarbonyl, CpCo(CO)_2_, is readily obtained by the reaction of Co_2_(CO)_8_ with cyclopentadiene^15^ or, less conveniently, by the reaction of cobaltocene, Cp_2_Co, with carbon monoxide under pressure.^16^ The binuclear Cp_2_Co_2_(CO)_3_ is an initial photolysis product of CpCo(CO)_2_ in a hydrocarbon solvent under mild conditions.^17^ However, it readily converts to the trinuclear Cp_3_Co_3_(CO)_3_ upon extended photolysis as initially reported by one of the current authors (RBK) in 1966.^18^ In addition, Cp_2_Co_2_(CO)_3_ readily loses a CO group to form the unsaturated Cp_2_Co_2_(CO)_2_ with a central Co=Co formal double bond.^12,19^ The tetranuclear derivative Cp_4_Co_4_(CO)_2_ is obtained by the pyrolysis of Cp_3_Co_3_(CO)_3_ at 130 °C in vacuum with liberation of 1 equiv of CpCo(CO)_2_. The rather unstable triplet state trinuclear Cp_3_Co_3_(CO)_2_ is obtained from the reaction of CpCo(CO)_2_ with 2 equiv of CpCo(C_2_H_4_)_2_ in a hexane solution.^13^

**Figure 1:**
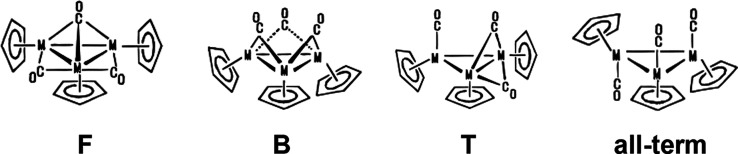
Four structures considered for the Cp_3_M_3_(CO)_3_ derivatives using the designations of Robbin et al.^20^

The trinuclear Cp_3_Co_3_(CO)_3_ is of interest because of its structural flexibility observed experimentally.^20^ The major isomer obtained from the photolysis of CpCo(CO)_2_ in toluene was shown by X-ray crystallography to have structure **F** (for face-bridging) (Figure 1) with one μ_3_-CO group bridging the Co_3_ triangle and the remaining μ-CO groups bridging different Co–Co edges.^21^ However, smaller quantities of a second isomer were isolated and shown to have structure **B** (for edge-bridging) with each edge of the central Co_3_ triangle bridged by a μ-CO group.^22^ The isomer **T** having one terminal CO group and two bridging groups has not been isolated as a stable species but is suggested to be present in solutions of isomer **F** based on the solution infrared spectrum of isomer **F**. No evidence is found for the existence of the all-terminal isomer **all-term** in the Cp_3_Co_3_(CO)_3_ system. However, the all-terminal isomer has been isolated for the analogous iridium species Cp_3_Ir_3_(CO)_3_ from the thermal decomposition of CpIr(*CO*)H_2_ and structurally characterized by X-ray diffraction.^23^

Extending the analogy between cyclopentadienylmetal carbonyls and binary metal carbonyls to nickel has the binuclear cyclopentadienylnickel carbonyl Cp_2_Ni_2_(CO)_2_, obtained from Cp_2_Ni and Ni(CO)_4_,^24^ as an analogue of the well-known Co_2_(CO)_8_. However, the trinuclear cyclopentadienylnickel carbonyl Cp_3_Ni_3_(CO)_2_ appears to be a thermodynamic sink in cyclopentadienylnickel carbonyl chemistry. This, at least initially, was very surprising since Cp_3_Ni_3_(CO)_2_ does not have a closed shell diamagnetic configuration but instead is a paramagnetic molecule with a single unpaired electron corresponding to a 19-electron configuration for one of the nickel atoms but delocalized over the central Ni_3_ triangle. Diamagnetic heterometallic derivatives of the type (CpCo)(CpNi)_2_(μ-CO)_2_, including species with methyl-substituted Cp rings, in which all three metal atoms have the favored 18-electron configuration, have been synthesized and characterized structurally by Dahl and co-workers.^25,26^ The cobalt carbonyl analogue of Cp_3_Ni_3_(CO)_2_, namely, Co_3_(CO)_10_, is not known as a stable paramagnetic molecule. However, the corresponding Co_3_(CO)_10_^–^ anion is obtained as its lithium salt^27^ by the reaction of LiCo(CO)_4_ with Co_2_(CO)_8_.

One objective of the current paper is to use modern density functional theory (DFT) methods to explore the energetic relationships between the four Cp_3_Co_3_(CO)_3_ isomers in Figure 1 including the experimentally observed **F** and **B** isomers isolated as stable species and characterized structurally by X-ray crystallography. In addition, the experimentally available species Cp_3_Co_3_(CO)_3_ and Cp_3_Ni_3_(CO)_2_ both have central nearly equilateral M_3_ triangles with the edges corresponding to formal single metal–metal bonds. Decarbonylation of such species might be expected to provide novel species having central M_3_ triangles with formal double or even triple bonds along the M–M edges. This paper reports DFT studies to explore such possibilities. In addition to predictions of formal M=M double bonds and M≡M triple bonds in unsaturated Cp_3_M_3_(CO)_*n*_ (M=Co, Ni; *n* = 2, 1, 0) species, unusual structures are predicted for the carbonyl-free Cp_3_Ni_3_ having agostic Ni–H–C interactions to Cp rings as well as Cp rings bridging Ni–Ni edges of the Ni_3_ triangle.

## 2 Theoretical Methods

DFT methods include electron correlation effects, which have been used extensively to model organometallic compounds.^28−34^ Among the many DFT methods, the Minnesota 2006 local functional (M06-L) is shown to be a good quality and relatively fast local density functional for a computational tool in organometallic chemistry and catalysis.^35^ Three DFT methods with different exchange-correlation (XC) energy functional, M06-L method,^36^ as well as the B3LYP^37,38^ and BP86^39,40^ methods were used to study the structures of Cp_3_M_3_(CO)_*n*_ (M = Co, Ni, *n* = 0–3) isomers in this paper. The geometries of all structures were fully optimized with double-ζ plus polarization (DZP) basis sets. For cobalt and nickel, the loosely contracted DZP basis set used the Wachters’ primitive sets augmented by two sets of p functions and one set of d functions and contracted following Hood et al. and designated as (14s11p6d/10s8p3d).^41,42^ The vibrational frequencies were determined at the same levels by evaluating analytically the second derivatives of the energy with respect to the nuclear coordinates. Because the numerical integration procedures used in existing DFT methods have significant limitations, we do not in general follow the imaginary eigenvector in search of another minimum when the predicted imaginary vibrational frequency is less than 50i cm^–1^. In such cases, there is a minimum of energy identical to or close to that of the stationary point in question.^43−45^

**Figure 2:**
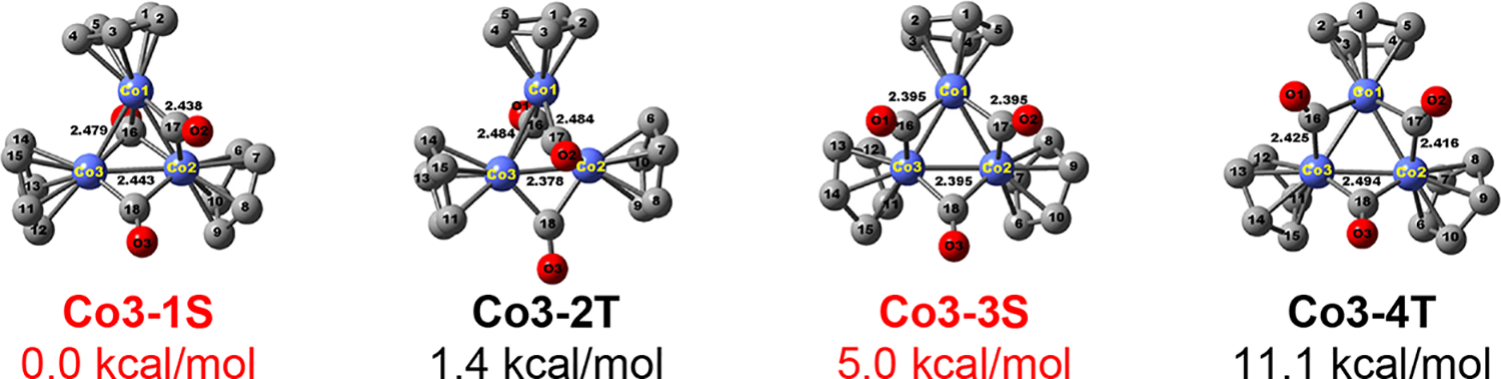
Four lowest-energy Cp_3_Co_3_(CO)_3_ structures. The two experimentally known structures are indicated in red.

**Figure 3:**
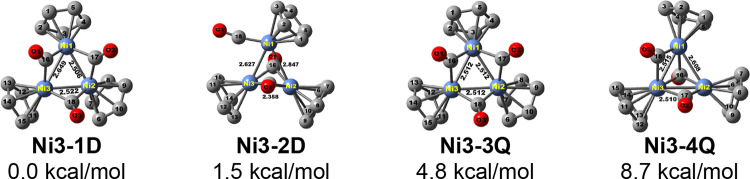
Four lowest-energy Cp_3_Ni_3_(CO)_3_ structures.

**Figure 4:**
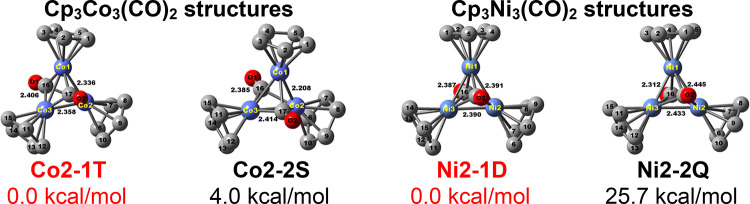
Two lowest-energy Cp_3_Co_3_(CO)_2_ structures and the two lowest-energy Cp_3_Ni_3_(CO)_2_ structures. The experimentally known Cp_3_Ni_3_(CO)_2_ structure is indicated in red.

**Figure 5:**
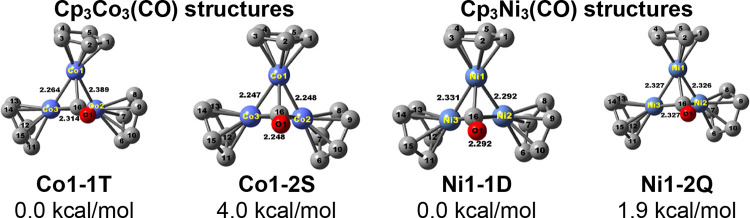
Two lowest-energy Cp_3_Co_3_(CO) structures and the two lowest energy Cp_3_Ni_3_(CO)_2_ structures.

**Figure 6:**
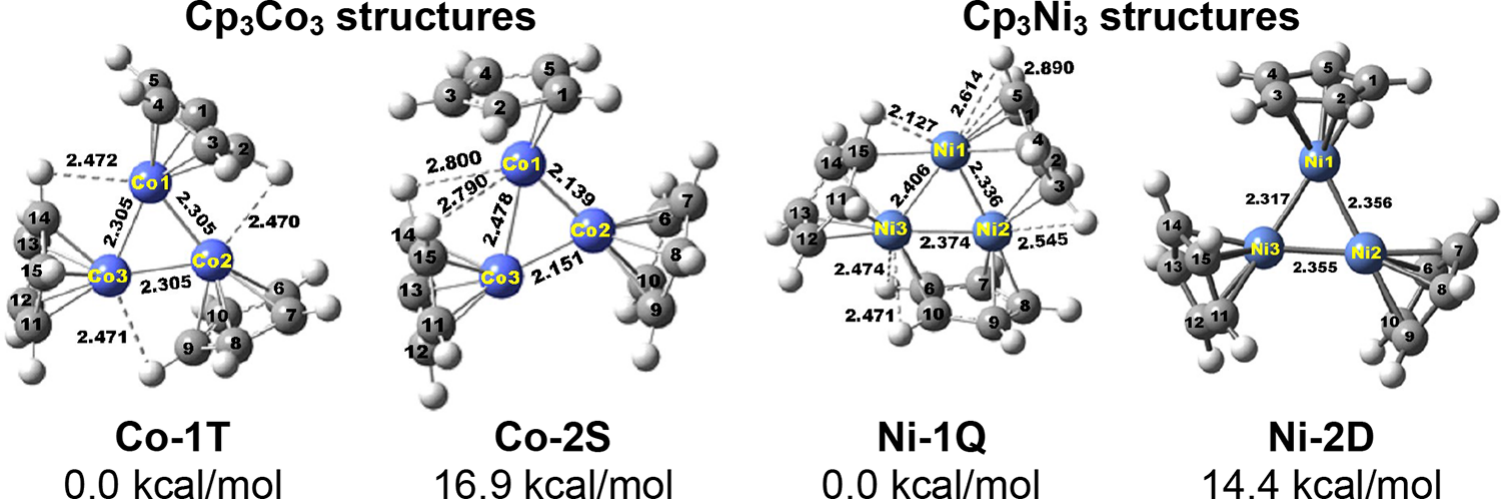
Two lowest-energy Cp_3_Co_3_ structures and the two lowest-energy Cp_3_Ni_3_ structures indicating the shortest distances between Cp ring hydrogen atoms and metal atoms.

All of the final optimized structures reported in this paper have only real vibrational frequencies unless otherwise indicated. The corresponding infrared intensities were evaluated analytically as well. All calculations were carried out in Gaussian 09 with tight optimizations and the ultrafine integration grid (99,590).^46^ Only the stationary point geometries of the energetically low-lying species in the Cp_3_M_3_(CO)_*n*_ (M = Co, Ni; *n* = 3, 2, 1, 0) systems predicted by M06-L are shown in Figures 2−6, with all listed interatomic distances given in Å. The results from the other two DFT methods are listed in the Supporting Information. For the vibrational frequencies, only the ν(CO) frequencies by BP86 are discussed. Comprehensive tables of the harmonic vibrational frequencies by BP86 are given in the Supporting Information.

## 3 Results and Discussion

### 3.1 Molecular Structures

#### 3.1.1 Cp_3_Co_3_(CO)_3_ and Cp_3_Ni_3_(CO)_3_

The search for the stable isomers of Cp_3_Co_3_(CO)_3_ and Cp_3_Ni_3_(CO)_3_ used the four types of starting structures depicted in Figure 1. Only type **B** and **F** structures were found among the low-energy structures (Figure 2 and Table 1), consistent with the experimental structures determined by X-ray crystallography as well as infrared spectroelectrochemistry and electron paramagnetic resonance (EPR) spectroscopy.^7^ The lowest-energy Cp_3_Co_3_(CO)_3_ structure is the singlet structure **Co3-1S**, with one face-bridging and two edge-bridging CO groups corresponding to isomer **F** in Figure 1. The Co–Co bond lengths of the edges with one face-bridging CO and one edge-bridging CO in **Co3-1S** are 2.44 Å. The Co–Co bond length of the edge without a bridging CO group is only slightly longer at 2.48 Å. These predicted Co–Co edge lengths in **Co3-1S** are very close to the experimental values determined by X-ray crystallography (Table 1).^21^ The triplet structure **Co3-2T**, lying only 1.4 kcal/mol in energy above **Co3-1S**, has a similar Cp_3_Co_3_(μ_3_-CO)(μ-CO)_2_ configuration.

**Table 1:** Four Lowest-Energy Cp_3_Co_3_(CO)_3_ Structures

structure	Δ*E*, kcal/mol	⟨*S*^2^⟩	Co–Co distances, Å	carbonyl configuration	ν(CO), cm^–1^
					BP86
**Co3-1S**	0.0		2.438, 2.443, 2.479	μ_3_-CO + 2 μ-CO	1708, 1802, 1839
Expt			2.438, 2.457, 2.519		1701, 1801, 1843
**Co3-2T**	1.4	2.12	2.378, 2.457, 2.519	μ_3_-CO + 2 μ-CO	1750, 1771, 1827
**Co3-3S**	5.0		3 × 2.395	3 μ-CO	1808, 1808, 1848
Expt			2.396, 2.408, 2.408		1784, 1784, 1839
**Co3-4T**	11.1	2.11	2.416, 2.425, 2.494	3 μ-CO	1806, 1819, 1858

The next Cp_3_Co_3_(CO)_3_ structures in terms of relative energy, namely, **Co3-3S** and **Co3-4T**, lying 5.0 and 11.1 kcal/mol above **Co3-1S**, respectively, approach idealized *C*_3*v*_ symmetry with each edge bridged by a CO group (Figure 2 and Table 1). The predicted Co–Co edge lengths of 2.395 Å in **Co3-3S** are essentially identical to the experimental values of 2.396, 2.408, and 2.408 Å determined by X-ray crystallography.^22^ Structures **Co3-3S** and **Co3-4T** thus correspond to isomer **B** in Figure 1. The closeness in energy of **Co3-1S** and **Co3-3S** is consistent with the degree of structural flexibility in this trinuclear system observed experimentally.^20^ Each of the three cobalt atoms in the singlet structures **Co3-1S** and **Co3-3S** has the favored 18-electron configuration by receiving five electrons from a neutral Cp group, two electrons from the two Co–Co bonds to adjacent cobalt atoms, and two electrons from a CO group. In the Cp_3_Co_3_(CO)_3_ derivatives, the edge-bridging ν(CO) frequencies range from 1806 to 1858 cm^–1^, whereas the face-bridging ν(CO) frequencies are significantly lower ranging from 1701 to 1750 cm^–1^ in accord with expectation.

Two doublet Cp_3_Ni_3_(CO)_3_ structures were found of similar energies (Figure 3 and Table 2). The lower-energy structure **Ni3-1D** is a type **B** (Figure 1) triply edge-bridged Cp_3_Ni_3_(μ-CO)_3_ structure similar to the experimentally known structure **Co3-3S** for Cp_3_Co_3_(CO)_3_. Lying only 1.5 kcal/mol in energy above **Ni3-1D** is a type **F** structure **Ni3-2D** analogous to the experimentally known structure **Co3-1S** with one face-bridging CO group and two edge-bridging CO groups. The quartet Cp_3_Ni_3_(CO)_3_ structures **Ni3-3Q** and **Ni3-4Q** corresponding to the doublet structures **Ni3-1D** and **Ni3-2D**, respectively, are also low-energy structures lying 4.8 and 8.7 kcal/mol above **Ni3-1D**. Since all three cobalt atoms in the singlet Cp_3_Co_3_(CO)_3_ structures have the favored 18-electron configuration, the corresponding Cp_3_Ni_3_(CO)_3_ structures must have 19-electron configurations for the electron-richer nickel atoms.

**Table 2:** Four Lowest-Energy Cp_3_Ni_3_(CO)_3_ Structures

structure	Δ*E*, kcal/mol	⟨*S*^2^⟩	Ni–Ni distances, Å	carbonyl configuration	ν(CO), cm^–1^
					BP86
Ni3-1D	0.0	0.78	2.552, 2.649, 2.506	μ_3_-CO + 2 μ-CO	1815, 1828, 1862
Ni3-2D	1.5	0.79	2.358, 2.627, 2.847	CO + 2 μ-CO	1730, 1838, 1870
Ni3-3Q	4.8	3.85	3 × 2.512	3 μ-CO	1840, 1840, 1872
Ni3-4Q	8.7	3.86	2.510, 2.515, 2.608	μ_3_-CO + 2 μ-CO	1760, 1839, 1860

#### 3.1.2 Cp_3_Co_3_(CO)_2_ and Cp_3_Ni_3_(CO)_2_

The optimized Cp_3_Co_3_(CO)_2_ and Cp_3_Ni_3_(CO)_2_ structures have a central M_3_ triangle bridged by a μ_3_-CO group on each side of the triangular face to give structures with a central M_3_C_2_ trigonal bipyramid having the metal atoms in equatorial positions and the CO carbon atoms in axial positions (Figure 4 and Table 3). The doublet Cp_3_Ni_3_(μ_3_-CO)_2_ structure **Ni2-1D** is known experimentally as a remarkably stable species for a paramagnetic cyclopentadienylmetal carbonyl.^24^ In accord with this remarkable stability, **Ni2-1D** lies a large 25.7 kcal/mol in energy below its quartet spin state isomer **Ni2-2Q**. The predicted 2.39 Å Ni–Ni distances with WBI values of 0.23 in the central equilateral Ni_3_ triangle of **Ni2-1D** are essentially identical to the experimental values of 2.39 Å as determined by X-ray crystallography.^25^ The spin density of the one unpaired electron in the doublet **Ni2-1D** is distributed equally among the three nickel atoms at 0.27 on each nickel atom reflecting its *C*_3*v*_ symmetry. In the high-energy quartet Cp_3_Ni_3_(μ_3_-CO)_2_ isomer **Ni2-2Q**, the central Ni_3_ triangle undergoes a Jahn–Teller distortion to give a slightly distorted isosceles triangle with two long Ni–Ni distances of 2.43 and 2.44 Å and one short Ni–Ni distance of 2.31 Å corresponding to WBI values of 0.32, 0.32, and 0.25, respectively.

**Table 3:** Lowest-Energy Cp_3_M_3_(CO)_2_ (M = Co, Ni) Structures

structure	Δ*E*, kcal/mol	⟨*S*^2^⟩	M–M distances, Å	carbonyl configuration	ν(CO), cm^–1^
					BP86
**Co2-1T**	0.0	2.11	2.336, 2.358, 2.406	2 μ_3_-CO	1713, 1736
Expt			3 × 2.370		1710
**Co2-2S**	4.0		2.208, 2.385, 2.414	2 μ_3_-CO	1719, 1743
**Ni2-1D**	0.0	0.78	2.387, 2.390, 2.391	2 μ_3_-CO	1748, 1774
Expt			3 × 2.389		1750
**Ni2-2Q**	25.7	3.85	2.312, 2.433, 2.445	2 μ_3_-CO	1759, 1796

For the cobalt derivative Cp_3_Co_3_(μ_3_-CO)_2_, the energy difference between the singlet and triplet structures is relatively small with the singlet structure **Co2-2S** lying 4.0 kcal/mol above **Co2-1T** (Figure 4 and Table 3). The lower energy of the triplet spin-state Cp_3_Co_3_(μ_3_-CO)_2_ isomer is consistent with its synthesis as a triplet state rather unstable molecule from CpCo(CO)_2_ and CpCo(C_2_H_4_)_2_ that has been structurally characterized by X-ray crystallography.^13^ Our calculations show that the central Co_3_ triangles in both **Co2-1T** and **Co2-2S** are distorted with much greater distortion in the singlet structure **Co2-2S**. Thus, in the triplet structure **Co2-1T**, the calculated Co–Co distances are 2.336, 2.358, and 2.406 Å with corresponding WBI values of 0.32, 0.33, and 0.41, respectively, corresponding to formal single bonds. A single Co–Co distance of 2.370 Å was found experimentally for all three Co–Co bonds in the Cp_3_Co_3_(μ_3_-CO)_2_ structure **Co2-1T** by X-ray crystallography^13^ corresponding exactly to the mean of the three calculated values.

The singlet Cp_3_Co_3_(μ_3_-CO)_2_ structure **Co2-2S** has one short Co=Co distance of 2.208 Å with a WBI of 0.54 that can be considered as a formal double bond as well as longer Co–Co distances of 2.385 and 2.414 Å with WBIs of 0.38 and 0.37 that can be considered as formal single bonds. Thus, loss of a bridging carbonyl group from the singlet tricarbonyl Cp_3_Co_3_(μ_3_-CO)(μ-CO)_2_ structure **Co3-1S** with 18-electron configurations for all three cobalt atoms to give the singlet dicarbonyl **Co2-2S** is balanced by increasing the bond order of one of the edges of the central Co_3_ triangle from single to double as reflected in one Co=Co edge becoming ∼0.2 Å shorter than the other two Co–Co edges. This allows each of the cobalt atoms in the dicarbonyl **Co2-2S** to retain the favored 18-electron configuration for a singlet spin-state structure.

The triplet structure **Co2-1T** has a spin density of 1.91 for the triplet spin state concentrated on one of the cobalt atoms with the other two cobalt atoms bearing essentially zero spin density. Structur**e Co2-1T** can be constructed from the stable known species Cp_2_Co_2_(μ-CO)_2_ with a formal Co=Co double bond and 18-electron cobalt configurations by adding a CpCo moiety across the Co=Co double bond. This reduces the original Co=Co double bond order in the Cp_2_Co_2_(μ-CO)_2_ moiety to a single Co–Co bond. However, the new Co–Co linkages from the CpCo moiety give that moiety a 16-electron configuration. In a high-spin system, this can account for the two unpaired electrons of the triplet spin state of **Co2-1T**. During this process, the two edge-bridging μ-CO groups in the binuclear Cp_2_Co_2_(μ-CO)_2_ moiety become face-bridging μ_3_-CO groups in the trinuclear structure.

#### 3.1.3 Cp_3_Co_3_(CO) and Cp_3_Ni_3_(CO)

All of the low-energy Cp_3_M_3_(CO) (M = Co, Ni) structures have a μ_3_-CO group bridging a central M_3_ triangle (Figure 5 and Table 4). For both cobalt and nickel, the same type of structure with different spin states is closely spaced in energy. Thus, the singlet Cp_3_Co_3_(μ_3_-CO) structure **Co1-2S** lies only 4.0 kcal/mol in energy above its triplet isomer **Co1-1T**. Similarly, the quartet Cp_3_Ni_3_(μ_3_-CO) structure **Ni1-2Q** lies only 1.9 kcal/mol in energy above its doublet isomer **Ni1-1D**. The singlet Cp_3_Co_3_(μ_3_-CO) structure **Co1-2S** has ideal *C*_3*v*_ symmetry with a central equilateral Co_3_ triangle with 2.248 Å edges short enough to suggest multiple bonding. A delocalized resonance hybrid having three equivalent canonical structures with two Co=Co double bonds and one Co–Co single bond for **Co1-2S** gives each cobalt atom the favored 18-electron configuration. The triplet Cp_3_Co_3_(μ_3_-CO) structure **Co1-1T**, however, appears to be distorted from ideal *C*_3*v*_ symmetry with Co=Co distances of 2.264 and 2.314 Å suggesting Co=Co double bonds and a longer Co–Co distance of 2.389 Å suggesting a Co–Co single bond. For the nickel systems, the quartet Cp_3_Ni_3_(μ_3_-CO) structure **Ni1-2Q** has ideal *C*_3*v*_ symmetry with equivalent Ni–Ni distances of 2.327 Å with WBIs of 0.32 in the central Ni_3_ triangle and equivalent spin densities of 0.75 on each nickel atom. The doublet Cp_3_Ni_3_(μ_3_-CO) structure **Ni1-1D** is only slightly distorted from *C*_3*v*_ symmetry with Ni–Ni distances of 2.29, 2.29, and 2.33 Å with WBIs of 0.38 falling within a fairly narrow 0.04 Å range. However, in **Ni1-1D**, the spin density is concentrated mainly on one of the three nickel atoms, which bears a spin density of 0.72 as compared with spin densities of only 0.02 on each of the other two nickel atoms.

**Table 4:** Lowest-Energy Cp_3_M_3_(CO) (M = Co, Ni) Structures

structure	Δ*E*, kcal/mol	⟨*S*^2^⟩	M–M distances, Å	carbonyl configuration	ν(CO), cm^–1^
					BP86
**Co1-1T**	0.0	2.97	2.264, 2.314, 2.389	μ_3_-CO	1738
**Co1-2S**	4.0		2.247, 2 × 2.248	μ_3_-CO	1739
**Ni1-1D**	0.0	0.87	2 × 2.292, 2.331	μ_3_-CO	1761
**Ni1-2Q**	1.9	3.85	3 × 2.327	μ_3_-CO	1748

#### 3.1.4 Cp_3_Co_3_ and Cp_3_Ni_3_

The lowest-energy structure for the carbonyl-free Cp_3_Co_3_, namely, **Co-1T**, has ideal *C*_3*v*_ symmetry with three equivalent Co–Co distances of 2.305 Å (Figure 6 and Table 5). The triplet spin density is also distributed evenly among the equivalent cobalt atoms with 0.70 on each atom. The locations of the Cp rings relative to the central Co_3_ triangle in **Co-1T** are distorted enough to bring one of the hydrogen atoms within 2.47 Å of a cobalt atom suggesting agostic C–H–Co bonding. The triplet spin state is greatly favored for Cp_3_Co_3_ since the singlet isomer **Co-2S** lies 16.7 kcal/mol in energy above the triplet isomer **Co-1T**. The central Co_3_ triangle in **Co-2S** has two short Co=Co distances of 2.139 and 2.151 Å with WBIs of 1.05 and 0.97 suggesting formal double bonds and one longer Co–Co distance of 2.478 Å with a WBI of 0.59 suggesting a formal single bond.

**Table 5:** Lowest-Energy Cp_3_M_3_ (M = Co, Ni) Structures

structure	Δ*E*, kcal/mol	⟨*S*^2^⟩	M–M distances, Å	structural features
**Co-1T**	0.0	2.23	3 × 2.305	Co–H(Cp): 3 × 2.47 Å
**Co-2S**	16.9		2.139, 2.151, 2.478	Co–H(Cp): 2 × 2.79 Å
**Ni-1Q**	0.0	3.80	2.336, 2.374, 2.406	bridging μ-η^3^,η^2^-Cp
**Ni-2D**	14.4	1.99	2.317, 2.355, 2.356	no short Ni–H(Cp)

The lowest-energy Cp_3_Ni_3_ structure is a rather unusual quartet structure **Ni-1Q** in which each Cp ring is a η^3^,η^2^ ligand bridging a Ni–Ni edge of the Ni_3_ triangle by forming a trihapto ligand to one nickel atom and a dihapto ligand to the other nickel atom (Figure 6 and Table 5). This is the only Cp_3_M_3_(CO)_*n*_ structure found in this work with bridging Cp ligands rather than terminal Cp ligands. Furthermore, one hydrogen atom in each of the bridging Cp ligands of **Ni-1Q** lies within 2.127 Å, indicating a C–H–Ni agostic interaction. The Ni–Ni distances of 2.336, 2.374, and 2.406 Å with WBIs of 0.32 are in a reasonable range for formal single bonds. Each nickel atom in **Ni-1Q** has a 19-electron configuration by receiving five electrons from portions of two bridging η^3^,η^2^-Cp ligands, two electrons from an agostic C–H–Ni interaction, and two electrons from Ni–Ni single bonds to adjacent nickel atoms. The quartet Cp_3_Ni_3_ structure **Ni-1Q** is favored significantly over its doublet isomer **Ni-2D** lying 14.4 kcal/mol in energy above **Ni-2D**. Structure **Ni-2D** has the usual terminal pentahapto Cp rings, no Ni–H distances short enough to indicate C–H–Ni agostic interactions, and fairly similar Ni–Ni distances of 2.317, 2.355, and 2.356 Å with WBIs of 0.57, 0.51, and 0.51 and in its central Ni_3_ triangle. One of the nickel atoms in **Ni-2D** has a spin density of 0.89 corresponding to the unpaired electron of its doublet spin state. The other two nickel atoms in **Ni-2D** have negligible spin densities with absolute values less than 0.07.

### 3.2 Thermochemistry

In order to check the viability of the Cp_3_M_3_(CO)_*n*_ (M = Co, Ni; *n* = 3, 2, 1) derivatives, the CO dissociation energies (Δ*E*_diss_) based on the lowest-energy structures were determined for the following processes:
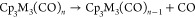


In addition, the following formulas were used to calculate the energies of formation (Δ*H*) and the Gibbs free energy (Δ*G*), where *E*_elec_, *E*_vib_, *E*_rot_, and *E*_transt_ correspond to the electronic energy, vibrational energy, rotational energy, and transition energy, respectively:
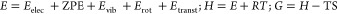


For all of these systems, the CO dissociation process is endothermic except for the trinickel tricarbonyl derivative Cp_3_Ni_3_(CO)_3_ for which CO dissociation to give the very stable dicarbonyl Cp_3_Ni_3_(μ_3_-CO)_2_ is clearly exothermic (see the red line in Table 6). The instability of Cp_3_Ni_3_(CO)_3_ toward CO loss clearly relates to the 19-electron configurations of its nickel atoms and the resulting need to shed one of its CO groups to bring the electron configurations of the nickel atoms down to 18 electrons or less.

**Table 6:** Energies (kcal/mol) for Carbonyl Dissociation of Cp_3_M_3_(CO)_*n*_ (M = Co, Ni; *n* = 3, 2, 1) Derivatives Based on the Global Minima for Each Structure by the M06L Method^a^

	Δ*E*, kcal/mol	Δ*H*_298_, kcal	Δ*G*_298_, kcal
Cp_3_Co_3_(CO)_3_ (**Co3-1S**) → Cp_3_Co_3_(CO)_2_ (**Co2-1T**) + CO	23.5	24.1	9.0
Cp_3_Co_3_(CO)_2_(**Co2-1T**) → Cp_3_Co_3_(CO) (**Co1-1T**) + CO	55.6	56.2	43.8
Cp_3_Co_3_(CO) (**Co1-1T**) → Cp_3_Co_3_ (**Co-1T**) + CO	62.7	63.3	54.1
Cp_3_Ni_3_(CO)_3_ (**Ni3-1D**) → Cp_3_Ni_3_(CO)_2_ (**Ni2-1D**) + CO	–7.4	–6.8	–14.3
Cp_3_Ni_3_(CO)_2_(**Ni2-1D**) → Cp_3_Ni_3_(CO) (**Ni1-1D**) + CO	42.7	43.3	27.2
Cp_3_Ni_3_(CO) (**Ni1-1D**) → Cp_3_Ni_3_ (**Ni-2Q**) + CO	47.6	48.2	38.8

a The single exothermic process is indicated in red.

### 3.3 Electron Paramagnetic Resonance

**Figure 7:**
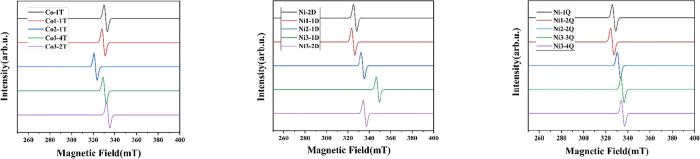
Simulated EPR spectra for triplet Cp_3_Co_3_(CO)_*n*_ (*n* = 3, 2, 1, 0), doublet, and quartet Cp_3_Ni_3_(CO)_*n*_ (*n* = 3, 2, 1, 0).

EPR has been shown to be an efficient and reliable tool to reveal the local structures of transition metals in their paramagnetic compounds. The EPR spectra of triplet Cp_3_Co_3_(CO)_*n*_ (*n* = 3, 2, 1, 0) and doublet and quartet Cp_3_Ni_3_(CO)_*n*_ (*n* = 3, 2, 1, 0) have been simulated by ORCA^47,48^ and EasySpin^49^ programs based on the optimized structures (Figure 7). The shapes of the simulated EPR spectra and the positions of resonant magnetic fields are quite close to each other except for **Ni3-1D**. The anisotropy g exhibits slight differences for the different coordination numbers (Table 7). Our calculated values of *g_x_*, *g_y_*, and *g_z_* of 2.02, 2.02, and 2.06, respectively, for the experimentally known and very stable **Ni2-1D** structure of Cp_3_Ni_3_(μ_3_-CO)_2_ are close to the corresponding experimental values of 2.02, 2.02, and 2.10 in the most recent reported EPR study.^50^

**Table 7:** Calculated g Factors and Hyperfine Structure Constants (in 10^–4^ cm^–1^) for Cp_3_M_3_(CO)_*n*_ (Cp = η^5^-C_5_H_5_); M = Co, Ni; *n* = 3, 2, 1, 0)

	*g_x_*	*g_y_*	*g_z_*	*A*_1_	*A*_2_	*A*_3_
Co-1T	2.01	2.02	2.11	0.31	0.80	4.35
Co1-1T	2.04	2.06	2.09	–0.32	0.70	1.81
Co2-1T	2.01	2.13	2.18	–0.13	–0.55	–1.81
Co3-4 T	2.02	2.06	2.08	–0.23	–0.45	1.18
Co3-2T	2.02	2.03	2.06	–0.46	–1.87	2.83
Ni-1Q	2.04	2.08	2.10	1.60	–3.18	–3.28
Ni1-2Q	2.08	2.08	2.09	–1.59	–1.71	4.71
Ni2-2Q	2.02	2.05	2.07	–1.70	–1.81	4.23
Ni3-3Q	2.02	2.02	2.04	–0.38	0.73	–4.39
Ni3-4Q	2.01	2.02	2.04	–2.34	–2.55	5.23
Ni-2D	2.03	2.03	2.17	–2.36	–3.08	19.30
Ni1-1D	2.01	2.11	2.14	–0.51	–0.80	1.34
Ni2-1D	2.02	2.02	2.06	1.68	–2.05	–2.54
Ni3-1D	1.81	2.01	2.03	0.27	–1.09	2.19
Ni3-2D	1.99	2.02	2.05	–1.94	–2.51	17.32

## 4 Summary

The small calculated energy separation of 5.0 kcal/mol between the two lowest-energy singlet Cp_3_Co_3_(μ_3_-CO)(μ-CO)_2_ and Cp_3_Co_3_(μ-CO)_3_ isomers accounts for the experimental results, indicating that both isomers are stable species that can be isolated separately and structurally characterized by X-ray crystallography. The corresponding Cp_3_Ni_3_(CO)_3_ species in the nickel system are not viable owing to exothermic CO dissociation to give the experimentally observed very stable Cp_3_Ni_3_(μ-CO)_2_, which is found to be the lowest-energy isomer by a substantial margin of ∼25 kcal/mol. In all of the low-energy Cp_3_M_3_(CO)_*n*_ (*n* = 2, 1) structures, all of the carbonyl groups are face-bridging or semi-face-bridging μ_3_-CO groups bonded to all three metal atoms of the M_3_ triangle. In the lowest-energy carbonyl-free Cp_3_M_3_ (M = Co, Ni) structures, the Cp rings are oriented to bring one of their hydrogen atoms into bonding distance to a metal atom leading to agostic C–H–M interactions. In addition, the lowest-energy Cp_3_Ni_3_ is the only structure among all of the low-energy Cp_3_M_3_(CO)_*n*_ (M = Co, Ni; *n* = 3, 2, 1, 0) structures in which each Cp ring bridges an M–M edge of the M_3_ triangle rather than functioning as a terminal pentahapto ligand to a single metal atom.

## Supporting Information Available

Energies, relative energies, and metal–metal distances for the Cp_3_M_3_(CO)_*n*_ (M = Co, Ni, *n* = 3, 2, 1, 0) structures by the B3LYP and BP86 methods; complete tables of metal–carbon and carbon–carbon distances (in Å) for the Cp_3_M_3_(CO)_*n*_ (M = Co, Ni, *n* = 3, 2, 1, 0) structures by the M06L, B3LYP, and BP86 methods; harmonic vibrational frequencies of the Cp_3_M_3_(CO)_*n*_ (M = Co, Ni, *n* = 3, 2, 1, 0) structures by the BP86 method; Wiberg bond indices for the M–M bonds in Cp_3_M_3_(CO)_*n*_ (M = Co, Ni, *n* = 3, 2, 1, 0); and spin densities for the M atoms in Cp_3_M_3_(CO)_*n*_ (M = Co, Ni, *n* = 3, 2, 1, 0) (PDF)
Coordinates (XYZ)

## Supplementary Material

ao3c02849_si_001.pdf

ao3c02849_si_002.xyz
