# Supplementary material for: Unraveling the Major Differences between the Trinuclear Cyclopentadienylmetal Carbonyl Chemistry of Cobalt and That of Nickel—A Theoretical Study
Source: ACS Omega. 2023 Jul 6;8(28):25392–400. doi: 10.1021/acsomega.3c02849 (PMC10357559; doi:10.1021/acsomega.3c02849)
Supplement: Supplementary file 1 — ao3c02849_si_001.pdf [file ao3c02849_si_001.pdf]

Unravelling the Major Differences Between the Trinuclear Cyclopentadienylmetal Carbonyl Chemistry of Cobalt and That of Nickel—A Theoretical Study

Yuexia Lin,[a] Hongyan Wang,[a]* R. Bruce King,[b]*

[a]*School of Physical Science and Technology, Southwest Jiaotong University*
Key Laboratory of Advanced Technologies of Materials, Ministry of Education of China, Chengdu 610031, China
and
[b]*Department of Chemistry and Center for Computational Chemistry*
University of Georgia, Athens, Georgia 30602, USA

E-mail: hongyanw@swjtu.edu.cn; rbking@chem.uga.edu

Suppporting Information

• Energies, relative energies, and metal-metal distances for the $Cp_3M_3(CO)_n$ (M=Co, Ni, n =3, 2, 1, 0) structures by the B3LYP and BP86 methods (Table S1-S5);
• Complete tables of metal-carbon and carbon-carbon distances (in Å) for the $Cp_3M_3(CO)_n$ (M=Co, Ni, n =3, 2, 1, 0) structures by the M06L, B3LYP and BP86 methods(Table S6-S10);
• Harmonic vibrational frequencies of the $Cp_3M_3(CO)_n$ (M=Co, Ni, n =3, 2, 1, 0) structures by the BP86 method (Tables S11- S30);
• Wiberg bond indices for the M-M bonds in $Cp_3M_3(CO)_n$ (M=Co, Ni, n =3, 2, 1, 0) (Table S31)
•Spin densities for the M atoms in $Cp_3M_3(CO)_n$ (M = Co, Ni, n = 3, 2, 1, 0) (Table S32)

Table S1. Co-Co bond distances (in Å), total energies (E in Hartree), zero point energy (ZPE in Hartree), relative energies with ZPE (ΔE in kcal/mol), relative energies (ΔE in kcal/mol), and spin expectation values ⟨S^2⟩ for the $Cp_3Co_3(CO)_3$ structures

	**Co3-1S-d**		**Co3-2T-d**		**Co3-3S-a**		**Co3-4T-a**	
	B3LYP	BP86	B3LYP	BP86	B3LYP	BP86	B3LYP	BP86
Co1-Co2	2.496	2.458	2.528	2.458	2.300	2.419	2.441	2.471
Co2- Co3	2.493	2.462	2.488	2.462	2.300	2.426	2.434	2.571
Co3-Co1	2.542	2.526	2.549	2.526	2.300	2.419	2.440	2.464
∠312	59.31	59.18	58.68	59.18	60.00	60.19	59.83	62.79
–Energy	5069.24186	5070.00084	5069.24552	5069.98969	5069.24324	5069.99581	5069.24476	5069.97528
ZPE	0.27973	0.27184	0.27757	0.26983	0.27985	0.27229	0.27722	0.26999
ΔE	0.00	0.00	-3.65	5.74	-0.79	3.44	-3.39	14.88
Imaginary Frequencies	no	no	no	16	no	no	57	24
$<S^2>$	---	---	2.29	2.04	---	---	2.21	2.03

Table S2. Ni-Ni bond distances (in Å), total energies (E in Hartree), zero point energy (ZPE in Hartree), relative energies with ZPE (ΔE in kcal/mol), relative energies (ΔE in kcal/mol), and spin expectation values ⟨S^2⟩ for the $Cp_3Ni_3(CO)_3$ structures

	**Ni3-1D-a**		**Ni3-2D-c**		**Ni3-3Q-a**		**Ni3-4Q-d**	
	B3LYP	BP86	B3LYP	BP86	B3LYP	BP86	B3LYP	BP86
Ni1-Ni2	2.551	2.603	3.271	2.534	2.557	2.554	2.688	2.702
Ni2-Ni3	2.557	2.540	2.564	2.460	2.567	2.557	2.635	2.556
Ni3-Ni1	2.850	2.703	2.564	2.459	2.563	2.556	2.659	2.567
∠312	56.19	57.18	50.37	59.03	60.18	60.06	59.05	57.98
–Energy	5445.88405	5446.63845	5445.87915	5446.632776	5445.88643	5446.61890	5445.87430	5446.61285
ZPE	0.27478	0.26757	0.27454	0.26676	0.27328	0.26608	0.27282	0.26584
ΔE	0.00	0.00	2.92	3.05	-2.43	11.33	4.89	14.98
Imaginary Frequencies	no	no	no	28	no	no	no	no
$<S^2>$	0.85	0.76	0.83	0.76	3.92	3.78	3.90	3.78

Table S3. Metal-metal distances (in Å), total energies (E in Hartree), zero point energy (ZPE in Hartree), relative energies with ZPE (ΔE in kcal/mol), relative energies (ΔE in kcal/mol), and spin expectation values ⟨S^2⟩ for the $Cp_3Co_3(CO)_2$ and $Cp_3Ni_3(CO)_2$ structures

	**Co2-1T**		**Co2-2S**		**Ni2-1D**		**Ni2-2Q**	
	B3LYP	BP86	B3LYP	**BP86**	B3LYP	BP86	B3LYP	BP86
M1-M2	2.459	2.207	2.206	2.206	2.393	2.441	2.514	2.454
M2- M3	2.459	2.445	2.430	2.430	2.382	2.434	2.501	2.459
M3-M1	2.426	2.454	2.418	2.418	2.314	2.420	2.349	2.325
∠312	60.46	63.01	63.21	63.21	60.79	60.10	61.78	61.86
–Energy	4955.89840	4956.61118	4955.87179	4956.61254	5332.55596	5333.30638	5332.52749	5333.25309
ZPE	0.26937	0.26132	0.27067	0.26236	0.26793	0.26083	0.26484	0.25690
ΔE	0.00	0.00	17.51	-0.20	0.00	0.00	15.93	30.97
Imaginary Frequencies	no	52	no	no	17	15	24	40
<S^2>	2.76	2.02	---	---	0.86	0.76	4.00	3.78

Table S4. Metal-metal distances (in Å), total energies (E in Hartree), zero point energy (ZPE in Hartree), relative energies with ZPE (ΔE in kcal/mol), relative energies (ΔE in kcal/mol), and spin expectation values ⟨S^2⟩ for the $Cp_3Co_3(CO)$ and $Cp_3Ni_3(CO)$ structures

	**Co1-1T**		**Co1-2S**		**Ni1-1D**		**Ni1-2Q**	
	B3LYP	BP86	B3LYP	BP86	B3LYP	BP86	B3LYP	BP86
M1-M2	2.506	2.248	2.241	2.300	2.393	2.293	2.393	2.345
M2- M3	2.327	2.243	2.243	2.296	2.382	2.304	2.382	2.346
M3-M1	2.3	2.247	2.247	2.363	2.314	2.363	2.314	2.345
∠312	57.74	59.87	59.97	58.99	60.79	59.28	60.79	60.00
–Energy	4841.54528	4843.17253	4841.53879	4843.18375	5219.17678	5219.89811	5219.17671	5219.88707
ZPE	0.25742	0.25180	0.26133	0.25323	0.25873	0.25158	0.25799	0.25129
ΔE	0.00	0.00	6.53	-6.14	0.00	0.00	-0.42	6.75
Imaginary Frequencies	no	18	no	no	10	no	19	27
<S^2>	3.44	2.1	---	---	1.44	0.78	3.97	3.78

Table S5. Metal-metal distances (in Å), total energies (E in Hartree), zero point energy (ZPE in Hartree), relative energies with ZPE (ΔE in kcal/mol), relative energies (ΔE in kcal/mol), and spin expectation values ⟨S^2⟩ for the Cp_3Co_3 and Cp_3Ni_3 structures

	**Co-1T**		**Co-2S**		**Ni-1Q**		**Ni-2D**	
	B3LYP	BP86	B3LYP	BP86	B3LYP	BP86	B3LYP	BP86
M1-M2	2.527	2.288	2.084	2.118	2.523	2.339	2.292	2.267
M2- M3	2.448	2.272	2.141	2.136	2.508	2.376	2.222	2.261
M3-M1	2.308	2.274	2.568	2.517	2.508	2.456	2.294	2.291
∠312	60.64	59.74	53.57	54.07	59.81	59.36	57.96	59.48
–Energy	4729.00905	4729.73216	4729.00766	4729.72528	5105.78071	5106.47099	5105.73311	5106.45328
ZPE	0.24774	0.24228	0.25199	0.24403	0.24726	0.24003	0.25002	0.24270
ΔE	0.00	0.00	3.54	5.42	0.00	0.00	31.60	12.79
Imaginary Frequencies	no	no	16	34	no	no	15	31
<S^2>	4.76	2.09	---	---	3.78	3.77	1.02	0.95

Table S6. Metal-carbon and carbon-carbon distances (in Å) for the $Cp_3Co_3(CO)_3$ structures by the M06L, B3LYP and BP86 methods

	**Co3-1S**			**Co3-2T**			**Co3-3S**			**Co3-4T**		
	M06L	B3LYP	BP86	M06L	B3LYP	BP86	M06L	B3LYP	BP86	M06L	B3LYP	BP86
Co1-C1	2.104	2.168	2.143	2.096	2.139	2.143	2.081	2.110	2.122	2.165	2.125	2.236
Co1-C2	2.067	2.113	2.101	2.112	2.132	2.101	2.117	2.110	2.122	2.167	2.114	2.245
Co1-C3	2.071	2.111	2.105	2.095	2.140	2.105	2.122	2.110	2.157	2.162	2.175	2.264
Co1-C4	2.077	2.117	2.107	2.117	2.174	2.107	2.055	2.110	2.122	2.165	2.134	2.244
Co1-C5	2.097	2.138	2.132	2.097	2.137	2.132	2.077	2.110	2.122	2.176	2.125	2.234
Co2-C6	2.106	2.158	2.161	2.134	2.233	2.161	2.055	2.110	2.098	2.103	2.101	2.124
Co2-C7	2.067	2.105	2.104	2.133	2.211	2.104	2.122	2.110	2.183	2.122	2.186	2.183
Co2-C8	2.080	2.119	2.111	2.097	2.160	2.111	2.117	2.110	2.180	2.091	2.171	2.187
Co2-C9	2.072	2.105	2.106	2.089	2.169	2.106	2.081	2.110	2.094	2.082	2.119	2.133
Co2-C10	2.116	2.161	2.164	2.131	2.212	2.164	2.077	2.110	2.097	2.095	2.120	2.126
Co3-C11	2.075	2.117	2.107	2.093	2.140	2.107	2.117	2.110	2.182	2.122	2.182	2.175
Co3-C12	2.087	2.134	2.125	2.082	2.124	2.125	2.122	2.110	2.179	2.130	2.178	2.188
Co3-C13	2.119	2.172	2.154	2.125	2.173	2.154	2.055	2.110	2.093	2.086	2.103	2.129
Co3-C14	2.075	2.117	2.111	2.122	2.140	2.111	2.077	2.110	2.098	2.080	2.117	2.125
Co3-C15	2.069	2.114	2.102	2.126	2.162	2.102	2.081	2.110	2.099	2.076	2.104	2.135
C1-C2	1.426	1.428	1.440	1.421	1.422	1.440	1.438	1.469	1.451	1.428	1.442	1.435
C2-C3	1.435	1.441	1.448	1.429	1.441	1.448	1.414	1.469	1.436	1.421	1.429	1.424
C3-C4	1.422	1.421	1.434	1.428	1.423	1.434	1.434	1.469	1.436	1.428	1.423	1.425
C4-C5	1.432	1.440	1.448	1.421	1.430	1.448	1.433	1.469	1.451	1.423	1.444	1.433
C5-C1	1.421	1.422	1.433	1.435	1.436	1.433	1.417	1.469	1.425	1.422	1.415	1.422
C6-C7	1.434	1.439	1.448	1.429	1.428	1.448	1.434	1.469	1.452	1.424	1.437	1.436
C7-C8	1.424	1.428	1.438	1.418	1.425	1.438	1.414	1.469	1.421	1.422	1.414	1.417
C8-C9	1.427	1.428	1.439	1.439	1.439	1.439	1.438	1.469	1.452	1.437	1.445	1.437
C9-C10	1.431	1.438	1.447	1.420	1.424	1.447	1.417	1.469	1.439	1.418	1.420	1.426
C10-C6	1.416	1.417	1.426	1.425	1.428	1.426	1.433	1.469	1.438	1.432	1.435	1.432
C11-C12	1.438	1.442	1.451	1.440	1.450	1.451	1.414	1.469	1.421	1.417	1.412	1.420
C12-C13	1.420	1.423	1.433	1.420	1.420	1.433	1.434	1.469	1.452	1.433	1.443	1.432
C13-C14	1.423	1.426	1.437	1.425	1.429	1.437	1.433	1.469	1.439	1.422	1.427	1.434
C14-C15	1.437	1.441	1.450	1.431	1.440	1.450	1.417	1.469	1.437	1.430	1.428	1.425
C15-C11	1.418	1.420	1.431	1.416	1.414	1.431	1.438	1.470	1.452	1.431	1.442	1.436
Co1-C16	1.958	1.951	1.936	1.850	1.847	1.936	1.890	1.901	1.884	2.032	1.893	2.083
Co2-C16	1.942	1.948	1.989	2.057	2.001	1.989	---	---	---	---	---	---
Co3-C16	1.949	1.920	1.936	2.088	2.186	1.936	1.892	1.901	1.886	1.824	1.880	1.814
C16-O1	1.206	1.204	1.218	1.198	1.194	1.218	1.186	1.258	1.199	1.181	1.184	1.174
Co1-C17	1.797	1.788	1.812	1.811	1.813	1.812	1.892	1.901	1.883	2.049	1.897	2.076
Co2-C17	2.011	2.050	1.973	2.112	2.022	1.973	1.890	1.901	1.887	1.817	1.885	1.819
C17-O2	1.187	1.182	1.200	1.194	1.184	1.200	1.186	1.258	1.199	1.180	1.183	1.174
Co2-C18	1.999	1.997	1.970	1.890	1.948	1.970	1.892	1.901	1.883	1.901	1.888	1.896
Co3-C18	1.807	1.809	1.816	1.875	1.856	1.816	1.890	1.901	1.884	1.892	1.885	1.898
C18-O3	1.187	1.183	1.200	1.187	1.180	1.200	1.186	1.258	1.199	1.187	1.183	1.184

Table S7. Metal-carbon and carbon-carbon distances (in Å) for the $Cp_3Ni_3(CO)_3$ structures by the M06L, B3LYP and BP86 methods

	**Ni3-1D**			**Ni3-2D**			**Ni3-3Q**			**Ni3-4Q**		
	M06L	B3LYP	BP86	M06L	B3LYP	BP86	M06L	B3LYP	BP86	M06L	B3LYP	BP86
Ni1-C1	2.115	2.235	2.160	2.204	2.168	2.107	2.187	2.220	2.221	2.139	2.207	2.174
Ni1-C2	2.152	2.229	2.170	2.224	2.169	2.108	2.199	2.227	2.235	2.170	2.204	2.175
Ni1-C3	2.179	2.154	2.212	2.227	2.250	2.110	2.187	2.229	2.228	2.202	2.231	2.215
Ni1-C4	2.167	2.157	2.202	2.197	2.209	2.145	2.170	2.225	2.208	2.207	2.263	2.244
Ni1-C5	2.159	2.237	2.194	2.184	2.141	2.122	2.172	2.219	2.207	2.164	2.238	2.217
Ni2-C6	2.172	2.217	2.228	2.108	2.141	2.122	2.168	2.204	2.206	2.179	2.245	2.230
Ni2-C7	2.206	2.271	2.267	2.127	2.168	2.107	2.187	2.231	2.229	2.147	2.209	2.185
Ni2-C8	2.215	2.271	2.245	2.111	2.169	2.109	2.198	2.234	2.237	2.153	2.201	2.167
Ni2-C9	2.163	2.215	2.165	2.183	2.250	2.108	2.187	2.211	2.220	2.182	2.227	2.200
Ni2-C10	2.110	2.164	2.131	2.175	2.210	2.145	2.170	2.198	2.205	2.196	2.261	2.236
Ni3-C11	2.181	2.244	2.239	2.122	2.172	2.111	2.196	2.236	2.236	2.182	2.230	2.245
Ni3-C12	2.175	2.225	2.211	2.142	2.224	2.106	2.186	2.234	2.228	2.196	2.231	2.243
Ni3-C13	2.122	2.142	2.139	2.196	2.278	2.164	2.170	2.214	2.207	2.223	2.248	2.251
Ni3-C14	2.125	2.165	2.148	2.159	2.278	2.163	2.172	2.207	2.206	2.224	2.252	2.256
Ni3-C15	2.183	2.246	2.232	2.118	2.224	2.104	2.187	2.216	2.221	2.195	2.235	2.246
C1-C2	1.438	1.449	1.453	1.418	1.432	1.432	1.428	1.445	1.445	1.422	1.425	1.434
C2-C3	1.414	1.420	1.431	1.425	1.441	1.450	1.420	1.435	1.431	1.429	1.437	1.445
C3-C4	1.430	1.430	1.438	1.426	1.411	1.437	1.426	1.436	1.440	1.419	1.425	1.434
C4-C5	1.424	1.434	1.446	1.420	1.447	1.435	1.427	1.444	1.442	1.423	1.427	1.435
C5-C1	1.420	1.417	1.428	1.430	1.423	1.449	1.419	1.429	1.431	1.430	1.433	1.444
C6-C7	1.420	1.426	1.434	1.423	1.423	1.449	1.426	1.443	1.441	1.428	1.432	1.441
C7-C8	1.426	1.428	1.438	1.424	1.432	1.431	1.419	1.429	1.431	1.423	1.426	1.437
C8-C9	1.419	1.426	1.433	1.436	1.441	1.450	1.428	1.444	1.445	1.429	1.435	1.443
C9-C10	1.430	1.433	1.447	1.409	1.411	1.437	1.419	1.435	1.432	1.422	1.427	1.437
C10-C6	1.429	1.433	1.439	1.437	1.447	1.434	1.427	1.439	1.441	1.422	1.426	1.434
C11-C12	1.424	1.426	1.437	1.429	1.433	1.438	1.420	1.430	1.431	1.424	1.429	1.434
C12-C13	1.420	1.425	1.434	1.426	1.425	1.448	1.426	1.441	1.440	1.426	1.432	1.443
C13-C14	1.438	1.447	1.452	1.418	1.428	1.426	1.427	1.440	1.442	1.419	1.424	1.430
C14-C15	1.416	1.417	1.430	1.431	1.425	1.447	1.419	1.432	1.431	1.425	1.431	1.441
C15-C11	1.428	1.435	1.440	1.425	1.433	1.439	1.429	1.445	1.445	1.425	1.429	1.438
Ni1-C16	1.892	1.900	1.886	2.188	1.999	1.937	1.949	1.936	1.936	2.011	1.944	1.956
Ni2-C16	---	---	---	1.910	2.000	1.933	---	---	---	2.013	1.963	1.965
Ni3-C16	1.895	1.899	1.897	1.949	1.985	1.989	1.969	1.938	1.936	2.074	2.399	2.188
C16-O1	1.184	1.185	1.196	1.192	1.198	1.218	1.176	1.189	1.190	1.190	1.179	1.203
Ni1-C17	1.911	1.879	1.886	1.885	1.799	1.816	1.968	1.930	1.936	1.888	1.958	1.905
Ni2-C17	1.931	1.977	1.917	1.905	2.130	1.969	1.950	1.943	1.935	2.060	2.000	1.976
C17-O2	1.179	1.173	1.193	1.186	1.170	1.200	1.176	1.189	1.190	1.175	1.169	1.190
Ni2-C18	1.946	1.980	1.923	1.794	1.799	1.812	1.967	1.935	1.937	1.889	1.971	1.910
Ni3-C18	1.902	1.877	1.903	2.727	2.128	1.975	1.950	1.934	1.935	2.063	1.999	1.973
C18-O3	1.179	1.173	1.192	1.163	1.170	1.200	1.176	1.190	1.190	1.175	1.168	1.190

Table S8. Metal-carbon and carbon-carbon distances (in Å) for the $Cp_3Co_3(CO)_2$ and $Cp_3Ni_3(CO)_2$ structures by the M06L, B3LYP and BP86 methods

	**Co2-1T**			**Co2-2S**			**Ni2-1D**			**Ni2-2Q**		
	M06L	B3LYP	BP86	M06L	B3LYP	BP86	M06L	B3LYP	BP86	M06L	B3LYP	BP86
M1-C1	2.063	2.109	2.098	2.040	2.074	2.074	2.127	2.145	2.173	2.153	2.207	2.183
M1-C2	2.084	2.133	2.123	2.036	2.079	2.079	2.119	2.177	2.168	2.136	2.189	2.165
M1-C3	2.086	2.155	2.136	2.081	2.122	2.122	2.115	2.159	2.166	2.136	2.186	2.163
M1-C4	2.080	2.136	2.101	2.037	2.081	2.081	2.117	2.176	2.169	2.156	2.208	2.184
M1-C5	2.057	2.109	2.094	2.039	2.073	2.073	2.123	2.171	2.170	2.161	2.214	2.192
M2-C6	2.182	2.293	2.108	2.068	2.093	2.093	2.123	2.200	2.166	2.151	2.205	2.193
M2-C7	2.135	2.278	2.090	2.056	2.081	2.081	2.125	2.196	2.163	2.154	2.207	2.192
M2-C8	2.132	2.230	2.097	2.056	2.081	2.081	2.123	2.189	2.166	2.152	2.199	2.187
M2-C9	2.192	2.220	2.090	2.068	2.093	2.093	2.116	2.191	2.165	2.172	2.215	2.205
M2-C10	2.213	2.256	2.135	2.097	2.130	2.130	2.116	2.193	2.165	2.150	2.198	2.186
M3-C11	2.082	2.136	2.122	2.078	2.114	2.114	2.125	2.145	2.179	2.133	2.191	2.166
M3-C12	2.061	2.112	2.128	2.091	2.123	2.123	2.124	2.163	2.177	2.146	2.201	2.183
M3-C13	2.060	2.113	2.121	2.090	2.122	2.122	2.120	2.178	2.172	2.156	2.207	2.192
M3-C14	2.087	2.131	2.126	2.091	2.123	2.123	2.115	2.159	2.165	2.150	2.199	2.185
M3-C15	2.083	2.151	2.121	2.078	2.114	2.114	2.118	2.175	2.168	2.134	2.188	2.163
C1-C2	1.426	1.431	1.434	1.441	1.455	1.455	1.426	1.430	1.430	1.427	1.430	1.443
C2-C3	1.428	1.429	1.427	1.423	1.436	1.436	1.427	1.425	1.432	1.426	1.432	1.438
C3-C4	1.427	1.428	1.430	1.423	1.436	1.436	1.427	1.442	1.431	1.428	1.431	1.443
C4-C5	1.427	1.432	1.436	1.442	1.455	1.455	1.427	1.420	1.431	1.424	1.429	1.438
C5-C1	1.432	1.435	1.430	1.418	1.432	1.432	1.426	1.441	1.431	1.424	1.430	1.437
C6-C7	1.420	1.424	1.440	1.435	1.452	1.452	1.426	1.428	1.432	1.422	1.426	1.435
C7-C8	1.437	1.430	1.425	1.423	1.434	1.434	1.426	1.429	1.432	1.428	1.433	1.443
C8-C9	1.422	1.432	1.438	1.435	1.452	1.452	1.427	1.432	1.430	1.424	1.429	1.437
C9-C10	1.422	1.429	1.430	1.424	1.436	1.436	1.427	1.427	1.432	1.424	1.429	1.438
C10-C6	1.426	1.429	1.425	1.424	1.436	1.436	1.427	1.432	1.430	1.429	1.434	1.443
C11-C12	1.425	1.433	1.430	1.427	1.440	1.440	1.426	1.443	1.429	1.428	1.431	1.443
C12-C13	1.434	1.434	1.431	1.427	1.441	1.441	1.426	1.420	1.430	1.424	1.430	1.437
C13-C14	1.424	1.431	1.432	1.427	1.440	1.440	1.427	1.439	1.431	1.424	1.428	1.438
C14-C15	1.427	1.431	1.429	1.427	1.440	1.440	1.427	1.428	1.431	1.429	1.432	1.443
C15-C11	1.428	1.427	1.433	1.429	1.442	1.442	1.427	1.427	1.430	1.425	1.430	1.438
M1-C16	1.879	1.869	2.004	2.073	2.049	2.049	1.942	1.862	1.947	2.042	2.071	2.070
M2-C16	2.287	2.540	2.057	1.978	2.008	2.008	1.941	2.319	1.955	1.973	1.955	1.947
M3-C16	1.889	1.866	1.839	1.850	1.850	1.850	1.942	1.845	1.937	2.074	2.142	2.061
C16-O1	1.199	1.193	1.200	1.203	1.215	1.215	1.198	1.190	1.193	1.188	1.180	1.201
M1-C17	1.881	1.866	2.004	2.073	2.047	2.047	1.945	1.862	1.949	2.078	2.072	2.071
M2-C17	2.281	2.559	2.060	1.978	2.006	2.006	1.944	2.447	1.937	1.928	1.953	1.944
M3-C17	1.880	1.867	1.837	1.851	1.852	1.852	1.944	1.862	1.957	2.113	2.136	2.066
C17-O2	1.198	1.193	1.199	1.203	1.215	1.215	1.198	1.190	1.193	1.187	1.180	1.201

Table S9. Metal-carbon and carbon-carbon distances (in Å) for the $Cp_3Co_3(CO)$ and $Cp_3Ni_3(CO)$ structures by the M06L, B3LYP and BP86 methods

	**Co1-1T**			**Co1-2S**			**Ni1-1D**			**Ni1-2Q**		
	M06L	B3LYP	BP86	M06L	B3LYP	BP86	M06L	B3LYP	BP86	M06L	B3LYP	BP86
M1-C1	2.110	2.174	2.117	2.085	2.140	2.111	2.086	2.145	2.114	2.140	2.145	2.171
M1-C2	2.123	2.187	2.103	2.071	2.128	2.142	2.118	2.177	2.144	2.135	2.177	2.163
M1-C3	2.089	2.153	2.119	2.087	2.142	2.139	2.103	2.159	2.142	2.139	2.159	2.171
M1-C4	2.069	2.149	2.080	2.049	2.095	2.152	2.115	2.176	2.146	2.120	2.176	2.150
M1-C5	2.065	2.131	2.073	2.047	2.093	2.160	2.122	2.171	2.160	2.122	2.171	2.150
M2-C6	2.185	2.250	2.123	2.085	2.135	2.136	2.113	2.200	2.140	2.141	2.200	2.171
M2-C7	2.140	2.195	2.078	2.047	2.092	2.132	2.111	2.196	2.132	2.122	2.196	2.150
M2-C8	2.149	2.210	2.079	2.050	2.097	2.129	2.106	2.189	2.125	2.121	2.189	2.150
M2-C9	2.208	2.278	2.119	2.086	2.141	2.145	2.117	2.191	2.144	2.140	2.191	2.171
M2-C10	2.239	2.307	2.108	2.071	2.126	2.154	2.123	2.193	2.157	2.135	2.193	2.163
M3-C11	2.122	2.181	2.122	2.087	2.144	2.118	2.100	2.145	2.116	2.140	2.145	2.171
M3-C12	2.070	2.120	2.077	2.049	2.101	2.159	2.108	2.163	2.162	2.122	2.163	2.150
M3-C13	2.081	2.139	2.079	2.047	2.101	2.149	2.120	2.178	2.150	2.123	2.178	2.150
M3-C14	2.105	2.166	2.123	2.085	2.139	2.136	2.093	2.159	2.139	2.140	2.159	2.171
M3-C15	2.138	2.202	2.109	2.072	2.129	2.147	2.122	2.175	2.145	2.134	2.175	2.163
C1-C2	1.418	1.420	1.443	1.430	1.433	1.445	1.428	1.430	1.446	1.427	1.430	1.441
C2-C3	1.432	1.440	1.444	1.429	1.434	1.431	1.420	1.425	1.430	1.426	1.425	1.441
C3-C4	1.423	1.427	1.431	1.420	1.423	1.454	1.439	1.441	1.453	1.423	1.442	1.436
C4-C5	1.433	1.434	1.459	1.443	1.446	1.429	1.415	1.420	1.431	1.432	1.420	1.448
C5-C1	1.432	1.441	1.434	1.420	1.423	1.449	1.436	1.441	1.446	1.423	1.441	1.436
C6-C7	1.430	1.433	1.432	1.420	1.423	1.442	1.427	1.428	1.442	1.422	1.428	1.436
C7-C8	1.427	1.434	1.457	1.443	1.445	1.441	1.427	1.429	1.441	1.432	1.429	1.448
C8-C9	1.428	1.432	1.433	1.420	1.423	1.443	1.429	1.432	1.443	1.423	1.432	1.436
C9-C10	1.419	1.425	1.444	1.429	1.433	1.437	1.423	1.427	1.437	1.426	1.427	1.441
C10-C6	1.419	1.428	1.443	1.430	1.433	1.441	1.428	1.432	1.441	1.427	1.432	1.441
C11-C12	1.431	1.439	1.433	1.420	1.422	1.450	1.439	1.443	1.448	1.424	1.443	1.436
C12-C13	1.433	1.438	1.459	1.443	1.444	1.428	1.417	1.420	1.428	1.431	1.420	1.448
C13-C14	1.425	1.429	1.432	1.420	1.421	1.454	1.434	1.439	1.454	1.423	1.439	1.436
C14-C15	1.428	1.436	1.443	1.430	1.432	1.432	1.428	1.428	1.431	1.427	1.428	1.441
C15-C11	1.419	1.423	1.443	1.429	1.432	1.443	1.420	1.427	1.444	1.426	1.427	1.441
M1-C16	2.014	2.011	1.972	1.961	1.968	1.865	1.870	1.863	1.878	1.944	1.862	1.932
M2-C16	2.172	2.359	1.958	1.961	1.964	2.105	2.136	2.319	2.098	1.944	2.319	1.932
M3-C16	1.835	1.805	1.952	1.961	1.957	1.874	1.871	1.845	1.865	1.944	1.845	1.932
C16-O1	1.196	1.192	1.212	1.201	1.193	1.211	1.198	1.190	1.211	1.197	1.190	1.210

Table S10. Metal-carbon and carbon-carbon distances (in Å) for the Cp_3Co_3 and Cp_3Ni_3 structures by the M06L, B3LYP and BP86 methods

	**Co-1T**			**Co-2S**			**Ni-1Q**			**Ni2-2D**		
	M06L	B3LYP	BP86	M06L	B3LYP	BP86	M06L	B3LYP	BP86	M06L	B3LYP	BP86
M1-C1	2.028	2.211	2.094	2.001	2.042	2.024	2.147	2.192	2.179	2.196	2.095	2.263
M1-C2	2.030	2.277	2.044	2.072	2.085	2.060	2.120	2.199	2.116	2.623	2.704	2.762
M1-C3	2.027	2.329	2.022	2.153	2.200	2.178	2.114	2.194	2.106	2.622	3.055	2.758
M1-C4	2.103	2.290	2.127	2.070	2.195	2.166	2.135	2.194	2.162	2.195	2.765	2.256
M1-C5	2.102	2.225	2.156	2.000	2.074	2.052	2.160	2.200	2.213	2.012	2.154	2.010
M2-C6	2.102	2.278	2.130	2.078	2.113	2.107	2.098	2.049	2.028	2.377	2.355	2.577
M2-C7	2.103	2.325	2.157	2.083	2.120	2.113	2.138	2.185	2.161	2.175	2.247	2.228
M2-C8	2.028	2.289	2.084	2.070	2.122	2.108	2.172	2.291	2.251	2.100	2.199	2.076
M2-C9	2.030	2.214	2.052	2.108	2.154	2.147	2.151	2.220	2.200	2.176	2.275	2.229
M2-C10	2.027	2.206	2.044	2.109	2.147	2.146	2.111	2.091	2.059	2.378	2.415	2.579
M3-C11	2.102	2.216	2.149	2.062	2.105	2.104	2.134	2.081	2.101	2.141	2.240	2.209
M3-C12	2.103	2.196	2.142	2.137	2.212	2.189	2.159	2.209	2.184	2.176	2.238	2.199
M3-C13	2.028	2.222	2.038	2.063	2.155	2.106	2.148	2.292	2.229	2.176	2.247	2.199
M3-C14	2.030	2.241	2.036	1.991	2.034	2.011	2.123	2.199	2.169	2.141	2.253	2.210
M3-C15	2.027	2.239	2.073	1.990	2.015	2.010	2.111	2.053	2.093	2.124	2.259	2.223
C1-C2	1.445	1.435	1.452	1.436	1.435	1.448	1.430	1.431	1.443	1.443	1.454	1.457
C2-C3	1.445	1.424	1.462	1.422	1.436	1.447	1.424	1.425	1.444	1.440	1.449	1.451
C3-C4	1.431	1.429	1.447	1.423	1.417	1.428	1.430	1.436	1.444	1.443	1.406	1.458
C4-C5	1.421	1.428	1.434	1.436	1.436	1.448	1.423	1.424	1.435	1.428	1.433	1.444
C5-C1	1.431	1.434	1.443	1.433	1.435	1.447	1.423	1.433	1.436	1.428	1.436	1.443
C6-C7	1.421	1.426	1.433	1.426	1.430	1.440	1.431	1.441	1.447	1.436	1.411	1.457
C7-C8	1.431	1.427	1.443	1.427	1.429	1.440	1.421	1.420	1.433	1.423	1.437	1.439
C8-C9	1.445	1.429	1.452	1.434	1.436	1.446	1.423	1.427	1.437	1.423	1.414	1.438
C9-C10	1.446	1.437	1.456	1.422	1.430	1.435	1.427	1.432	1.439	1.436	1.457	1.457
C10-C6	1.431	1.430	1.447	1.433	1.436	1.446	1.427	1.442	1.460	1.445	1.459	1.457
C11-C12	1.421	1.431	1.433	1.421	1.426	1.435	1.424	1.432	1.443	1.415	1.414	1.427
C12-C13	1.431	1.435	1.445	1.421	1.422	1.434	1.424	1.428	1.436	1.427	1.437	1.442
C13-C14	1.445	1.425	1.460	1.439	1.436	1.452	1.426	1.420	1.435	1.415	1.414	1.427
C14-C15	1.445	1.441	1.455	1.445	1.444	1.457	1.427	1.440	1.444	1.450	1.456	1.466
C15-C11	1.431	1.427	1.444	1.440	1.439	1.452	1.429	1.443	1.447	1.450	1.454	1.466

Table S11. Harmonic vibrational frequencies (in cm^{-1}) and infrared intensities (in parentheses, in km/mol) for the Co3-1S structure by the BP86 method

A	28(1)	297(11)	562(5)	846(4)	1107(4)	1839(589)
	29(0)	311(4)	566(1)	856(1)	1109(1)	3166(0)
	40(0)	322(8)	579(41)	863(2)	1221(0)	3166(0)
	50(0)	335(1)	668(229)	868(3)	1223(0)	3169(0)
	77(0)	340(0)	765(29)	871(0)	1223(0)	3170(0)
	104(0)	362(3)	771(10)	879(1)	1348(1)	3171(0)
	110(1)	380(3)	774(46)	980(7)	1350(4)	3171(0)
	116(0)	400(2)	780(14)	981(7)	1351(2)	3179(2)
	130(0)	447(4)	782(60)	983(6)	1354(2)	3180(3)
	139(0)	450(3)	798(30)	997(13)	1356(4)	3181(0)
	146(0)	455(45)	802(16)	998(2)	1357(6)	3182(1)
	183(0)	462(13)	804(1)	999(10)	1396(3)	3183(0)
	189(1)	489(10)	806(13)	1035(1)	1396(8)	3188(0)
	199(1)	504(11)	807(5)	1036(1)	1397(2)	3192(2)
	214(1)	527(17)	811(1)	1038(4)	1413(2)	3193(0)
	226(1)	544(9)	813(16)	1038(1)	1415(3)	3196(1)
	236(1)	552(1)	813(22)	1040(1)	1419(2)	
	250(0)	556(5)	816(4)	1042(0)	1708(384)	
	296(7)	559(1)	827(7)	1106(5)	1802(247)	

Table S12. Harmonic vibrational frequencies (in cm^{-1}) and infrared intensities (in parentheses, in km/mol) for the Co3-2T structure by the BP86 method

A	-16(0)	286(8)	562(4)	852(1)	1108(3)	1827(520)
	13(0)	297(2)	564(0)	854(0)	1109(2)	3161(0)
	38(0)	298(8)	569(1)	855(1)	1222(0)	3162(0)
	44(1)	303(9)	614(134)	865(1)	1223(0)	3170(0)
	52(1)	309(2)	754(55)	865(1)	1224(0)	3171(0)
	98(0)	319(2)	765(23)	877(8)	1343(13)	3172(0)
	105(1)	356(1)	777(72)	982(2)	1346(0)	3173(0)
	111(0)	386(3)	778(40)	983(11)	1351(0)	3175(2)
	115(0)	391(7)	785(39)	984(11)	1351(0)	3175(2)
	125(1)	396(1)	789(5)	996(10)	1352(0)	3183(0)
	141(1)	405(13)	799(12)	997(5)	1354(1)	3184(0)
	147(1)	439(47)	800(31)	999(9)	1397(3)	3185(1)
	161(3)	487(14)	802(8)	1034(4)	1398(1)	3186(1)
	166(8)	492(7)	805(4)	1037(1)	1398(4)	3186(0)
	195(0)	527(4)	809(7)	1037(1)	1414(0)	3196(1)
	200(0)	538(44)	810(3)	1038(0)	1415(2)	3197(1)
	211(0)	548(4)	811(2)	1040(0)	1415(2)	
	247(0)	554(24)	812(5)	1041(2)	1750(639)	
	275(1)	556(19)	816(4)	1107(3)	1771(110)	

Table S13. Harmonic vibrational frequencies (in cm-1) and infrared intensities (in parentheses, in km/mol) for the Co3-3S structure by the BP86 method

A	18(0)	322(5)	563(1)	854(6)	1107(3)	1848(914)
	43(0)	322(6)	578(6)	858(1)	1108(4)	3165(0)
	49(0)	334(0)	625(133)	865(1)	1220(0)	3166(0)
	70(0)	337(0)	626(129)	867(7)	1223(0)	3166(0)
	72(0)	355(0)	737(17)	869(10)	1225(0)	3167(0)
	100(0)	368(0)	748(11)	879(1)	1346(11)	3168(0)
	106(0)	382(0)	750(37)	972(3)	1350(1)	3168(2)
	116(0)	419(0)	786(61)	975(9)	1350(0)	3178(1)
	122(0)	420(5)	787(36)	981(5)	1357(16)	3179(2)
	132(0)	472(12)	790(35)	1000(7)	1358(9)	3179(4)
	133(0)	476(14)	797(0)	1001(14)	1364(7)	3184(1)
	137(0)	480(4)	805(16)	1004(5)	1388(4)	3187(0)
	190(0)	500(34)	806(11)	1029(8)	1390(9)	3187(1)
	191(0)	502(35)	808(3)	1035(0)	1391(3)	3192(2)
	218(0)	550(3)	809(1)	1037(1)	1423(4)	3194(1)
	236(0)	553(0)	811(11)	1039(0)	1425(1)	3195(4)
	245(0)	555(0)	815(3)	1040(1)	1426(2)	
	247(0)	556(2)	818(3)	1042(2)	1808(337)	
	292(6)	561(1)	837(13)	1106(3)	1808(334)	

Table S14. Harmonic vibrational frequencies (in cm-1) and infrared intensities (in parentheses, in km/mol) for the Co3-4T structure by the BP86 method

A	-24(0)	284(1)	557(15)	839(2)	1108(3)	1858(809)
	2(0)	287(4)	559(0)	849(5)	1110(3)	3163(0)
	35(0)	295(5)	573(27)	859(1)	1221(0)	3165(0)
	55(0)	310(0)	610(113)	864(3)	1223(0)	3167(0)
	60(0)	316(6)	737(30)	864(4)	1223(0)	3167(0)
	90(0)	318(3)	750(38)	868(5)	1347(4)	3168(0)
	91(1)	326(1)	770(6)	980(4)	1349(5)	3169(1)
	103(1)	372(13)	774(41)	982(8)	1350(0)	3178(2)
	107(0)	375(7)	781(54)	989(8)	1352(1)	3179(1)
	115(1)	447(14)	785(47)	991(9)	1355(5)	3179(2)
	120(0)	469(21)	787(25)	996(9)	1356(6)	3181(1)
	134(0)	486(35)	792(33)	998(10)	1395(2)	3185(2)
	146(0)	495(3)	800(8)	1030(4)	1396(2)	3185(0)
	169(0)	503(21)	806(5)	1035(2)	1405(2)	3193(2)
	202(1)	527(45)	808(9)	1036(3)	1413(2)	3193(1)
	205(1)	546(2)	810(2)	1038(1)	1414(1)	3193(2)
	218(0)	549(6)	811(4)	1039(2)	1416(2)	
	238(1)	551(4)	813(4)	1042(1)	1806(380)	
	277(7)	555(25)	817(5)	1107(2)	1819(388)	

Table S15. Harmonic vibrational frequencies (in cm^{-1}) and infrared intensities (in parentheses, in km/mol) for the Ni3-1D structure by the BP86 method

A	28(0)	233(2)	559(1)	843(2)	1107(0)	1862(831)
	36(0)	240(1)	567(1)	844(6)	1108(1)	3163(0)
	48(0)	254(1)	582(62)	848(7)	1221(0)	3163(0)
	53(0)	268(5)	596(79)	850(2)	1223(0)	3165(0)
	60(0)	281(17)	739(47)	855(0)	1225(0)	3165(0)
	70(1)	300(10)	748(27)	858(1)	1316(8)	3165(0)
	81(0)	310(12)	757(30)	981(13)	1339(1)	3167(1)
	93(0)	334(5)	760(22)	983(7)	1340(13)	3176(1)
	101(0)	370(12)	764(108)	988(12)	1343(15)	3178(1)
	102(0)	409(13)	770(77)	992(8)	1344(2)	3178(2)
	105(0)	413(1)	780(40)	996(9)	1350(4)	3179(2)
	108(0)	434(3)	786(8)	1001(7)	1397(3)	3182(1)
	113(1)	436(3)	791(17)	1023(3)	1400(4)	3183(1)
	132(0)	460(17)	794(9)	1034(4)	1404(3)	3190(2)
	176(0)	508(5)	808(0)	1035(4)	1411(1)	3192(3)
	191(1)	524(2)	809(16)	1036(7)	1418(1)	3192(2)
	200(0)	538(24)	811(3)	1038(1)	1422(1)	
	205(2)	548(8)	811(6)	1040(1)	1815(316)	
	216(1)	558(4)	817(0)	1107(0)	1828(280)	

Table S16. Harmonic vibrational frequencies (in cm^{-1}) and infrared intensities (in parentheses, in km/mol) for the Ni3-2D structure by the BP86 method

A	-29(0)	216(0)	560(6)	839(12)	1107(1)	1870(570)
	16(0)	225(1)	568(0)	849(1)	1109(0)	3164(1)
	23(0)	234(8)	576(109)	851(1)	1219(0)	3165(0)
	40(1)	253(3)	593(41)	852(1)	1222(0)	3165(0)
	44(0)	262(0)	727(9)	856(3)	1223(0)	3165(0)
	79(0)	305(22)	743(4)	857(3)	1331(3)	3166(0)
	83(0)	307(26)	748(97)	972(3)	1331(2)	3167(0)
	93(1)	331(1)	755(106)	975(15)	1335(15)	3176(1)
	96(1)	372(25)	759(58)	983(10)	1342(39)	3177(1)
	107(0)	397(3)	765(71)	996(9)	1346(0)	3180(0)
	109(0)	407(5)	790(12)	997(7)	1350(17)	3181(0)
	125(1)	417(7)	790(33)	1001(19)	1388(0)	3182(1)
	127(1)	427(20)	799(9)	1027(3)	1392(6)	3183(1)
	166(1)	439(5)	803(2)	1031(3)	1400(2)	3191(2)
	172(1)	447(1)	804(1)	1033(5)	1422(1)	3192(6)
	185(5)	527(23)	805(13)	1034(10)	1424(0)	3192(0)
	192(1)	538(24)	808(7)	1037(4)	1426(1)	
	208(0)	547(1)	813(1)	1039(3)	1730(318)	
	214(1)	558(16)	814(1)	1106(0)	1838(164)	

Table S17. Harmonic vibrational frequencies (in cm^{-1}) and infrared intensities (in parentheses, in km/mol) for the Ni3-3Q structure by the BP86 method

A	15(0)	203(2)	560(1)	838(1)	1108(0)	1872(947)
	17(0)	218(1)	560(1)	839(1)	1109(1)	3161(0)
	38(0)	228(0)	568(4)	841(0)	1223(0)	3162(0)
	52(0)	240(1)	568(4)	848(0)	1223(0)	3162(0)
	53(0)	241(1)	736(39)	850(1)	1226(0)	3163(0)
	73(0)	293(6)	741(37)	850(1)	1336(0)	3163(1)
	85(0)	294(6)	753(27)	983(14)	1339(6)	3164(0)
	90(0)	311(4)	760(97)	984(14)	1339(7)	3175(1)
	91(0)	355(0)	760(100)	985(1)	1345(4)	3175(2)
	101(0)	369(22)	764(45)	996(22)	1347(9)	3176(2)
	104(0)	369(22)	766(25)	997(4)	1347(9)	3179(1)
	106(0)	401(3)	773(22)	998(4)	1399(3)	3180(1)
	137(0)	402(3)	773(22)	1032(0)	1399(3)	3180(2)
	137(0)	409(2)	808(0)	1035(1)	1399(0)	3189(2)
	170(0)	456(1)	810(2)	1036(2)	1418(2)	3189(2)
	184(0)	513(39)	810(2)	1037(1)	1418(2)	3190(2)
	189(0)	514(39)	810(0)	1037(4)	1418(1)	
	189(0)	554(0)	813(2)	1037(4)	1840(313)	
	203(2)	559(0)	813(2)	1108(0)	1840(312)	

Table S18. Harmonic vibrational frequencies (in cm^{-1}) and infrared intensities (in parentheses, in km/mol) for the Ni3-4Q structure by the BP86 method

A	23(0)	202(2)	560(0)	845(2)	1108(0)	1860(618)
	25(0)	217(0)	563(1)	846(1)	1110(1)	3164(0)
	33(0)	220(2)	567(3)	848(0)	1223(0)	3164(0)
	36(0)	224(1)	568(7)	850(1)	1224(0)	3165(0)
	58(0)	231(0)	749(18)	850(1)	1224(0)	3165(0)
	82(0)	286(10)	754(63)	853(2)	1335(0)	3166(0)
	85(0)	301(4)	755(102)	984(7)	1337(4)	3166(0)
	90(0)	319(10)	759(78)	985(9)	1338(0)	3178(2)
	105(0)	355(15)	760(29)	986(12)	1344(2)	3178(1)
	109(0)	361(4)	772(15)	994(13)	1344(2)	3179(2)
	120(0)	369(5)	773(48)	996(8)	1345(4)	3180(1)
	127(0)	377(14)	775(15)	996(13)	1400(2)	3180(1)
	137(0)	396(1)	777(35)	1032(0)	1402(3)	3183(0)
	138(0)	404(2)	808(1)	1033(0)	1403(2)	3191(2)
	163(0)	433(9)	809(2)	1034(1)	1412(1)	3191(3)
	168(1)	483(17)	809(3)	1036(2)	1414(2)	3192(1)
	174(0)	508(54)	812(2)	1037(1)	1416(2)	
	194(2)	557(3)	812(0)	1039(0)	1760(340)	
	200(0)	558(1)	814(0)	1107(0)	1839(227)	

Table S19. Harmonic vibrational frequencies (in cm^{-1}) and infrared intensities (in parentheses, in km/mol) for the Co2-1T structure by the BP86 method

A	-52(0)	312(8)	568(43)	858(2)	1106(3)	3164(0)
	23(0)	315(10)	769(38)	860(0)	1222(0)	3164(0)
	33(0)	327(1)	775(40)	862(1)	1223(0)	3165(0)
	37(0)	340(4)	781(27)	863(1)	1224(0)	3165(0)
	67(0)	358(1)	785(23)	984(6)	1343(0)	3165(0)
	80(1)	373(3)	786(5)	985(15)	1345(0)	3166(0)
	90(0)	380(2)	790(19)	986(6)	1346(0)	3177(0)
	116(0)	387(0)	793(24)	992(7)	1348(2)	3177(1)
	128(0)	388(0)	796(3)	993(10)	1348(4)	3178(2)
	145(0)	432(14)	800(4)	994(11)	1351(1)	3178(1)
	177(0)	462(5)	802(0)	1034(0)	1395(0)	3179(2)
	185(1)	481(1)	807(4)	1034(0)	1396(4)	3179(2)
	191(0)	537(78)	808(1)	1035(0)	1400(2)	3189(2)
	196(1)	554(0)	809(5)	1036(0)	1406(1)	3189(1)
	218(0)	555(0)	811(11)	1037(0)	1407(2)	3190(1)
	227(0)	558(0)	813(1)	1039(0)	1408(3)	
	279(6)	558(0)	848(1)	1104(7)	1713(681)	
	303(0)	565(0)	850(0)	1104(4)	1736(3)	

Table S20. Harmonic vibrational frequencies (in cm^{-1}) and infrared intensities (in parentheses, in km/mol) for the Co2-2S structure by the BP86 method

A	7(0)	343(3)	622(124)	853(4)	1106(4)	3164(0)
	20(0)	345(1)	767(7)	855(1)	1220(0)	3164(0)
	41(0)	357(0)	773(33)	857(4)	1221(0)	3164(1)
	43(2)	359(0)	782(55)	863(0)	1222(0)	3165(0)
	72(0)	370(7)	783(43)	973(7)	1342(26)	3165(0)
	86(1)	373(7)	787(6)	977(9)	1343(0)	3167(0)
	116(0)	403(0)	791(1)	988(10)	1347(0)	3176(0)
	124(0)	406(3)	793(17)	989(10)	1350(0)	3177(2)
	129(3)	438(0)	794(13)	996(6)	1353(3)	3177(1)
	149(1)	447(8)	798(1)	997(13)	1353(0)	3179(1)
	186(4)	482(17)	801(5)	1027(5)	1388(2)	3179(1)
	194(0)	498(5)	808(1)	1035(0)	1393(5)	3180(3)
	196(0)	545(4)	809(2)	1035(1)	1402(2)	3188(1)
	203(0)	550(0)	812(0)	1036(0)	1404(2)	3189(2)
	220(0)	552(4)	815(5)	1038(1)	1412(2)	3190(1)
	241(0)	560(0)	815(10)	1038(0)	1413(2)	
	293(3)	566(0)	846(0)	1102(10)	1719(683)	
	314(10)	566(2)	851(1)	1103(6)	1743(3)	

Table S21. Harmonic vibrational frequencies (in cm^{-1}) and infrared intensities (in parentheses, in km/mol) for the Ni2-1D structure by the BP86 method

A	-15(0)	265(7)	581(57)	848(1)	1106(1)	3163(0)
	7(0)	279(0)	761(48)	852(1)	1221(0)	3164(0)
	17(0)	287(0)	763(73)	856(0)	1222(0)	3165(0)
	53(0)	289(0)	763(50)	859(0)	1222(0)	3165(0)
	56(0)	355(21)	771(45)	987(3)	1338(0)	3165(0)
	90(1)	356(20)	775(7)	987(5)	1339(0)	3165(0)
	111(0)	380(0)	776(12)	988(14)	1339(0)	3177(0)
	112(0)	403(0)	778(31)	989(19)	1340(0)	3178(3)
	125(0)	406(0)	780(30)	989(7)	1342(0)	3178(3)
	162(4)	460(6)	781(24)	990(18)	1347(0)	3179(0)
	163(4)	464(0)	808(0)	1032(0)	1403(0)	3180(0)
	181(0)	543(75)	809(0)	1033(1)	1403(2)	3180(1)
	191(0)	544(75)	810(0)	1033(0)	1404(1)	3190(3)
	192(0)	559(0)	811(3)	1035(0)	1404(3)	3190(3)
	198(0)	564(0)	812(4)	1036(0)	1404(2)	3191(0)
	201(0)	566(0)	817(0)	1038(0)	1406(2)	
	261(2)	571(1)	844(0)	1105(1)	1748(669)	
	263(0)	578(52)	847(0)	1105(1)	1774(1)	

Table S22. Harmonic vibrational frequencies (in cm^{-1}) and infrared intensities (in parentheses, in km/mol) for the Ni2-2Q structure by the BP86 method

A	-40(1)	229(0)	566(0)	842(2)	1106(0)	3161(0)
	18(3)	238(9)	747(51)	846(1)	1221(0)	3162(0)
	24(0)	247(0)	754(86)	847(0)	1222(0)	3163(0)
	24(0)	256(1)	755(28)	849(0)	1223(0)	3163(0)
	49(0)	264(1)	756(41)	983(7)	1330(17)	3164(1)
	60(0)	274(10)	761(5)	984(12)	1334(2)	3165(0)
	74(0)	302(3)	764(65)	985(15)	1337(0)	3175(2)
	84(0)	323(10)	766(0)	989(13)	1338(0)	3177(0)
	92(7)	342(1)	766(41)	991(7)	1341(1)	3177(1)
	105(0)	347(5)	770(6)	992(17)	1344(2)	3178(1)
	115(0)	357(6)	805(0)	1028(6)	1399(0)	3178(0)
	147(1)	416(13)	806(4)	1031(1)	1400(5)	3179(3)
	152(1)	477(28)	810(1)	1032(1)	1402(1)	3188(3)
	156(0)	557(0)	811(2)	1034(0)	1407(1)	3189(3)
	166(1)	558(0)	812(0)	1035(0)	1408(1)	3190(1)
	171(0)	560(0)	814(1)	1035(0)	1409(2)	
	194(2)	561(0)	835(0)	1104(1)	1759(717)	
	227(2)	566(0)	838(0)	1105(0)	1796(1)	

Table S23. Harmonic vibrational frequencies (in cm^{-1}) and infrared intensities (in parentheses, in km/mol) for the Co1-1T structure by the BP86 method

A	-18(0)	307(5)	764(31)	850(0)	1221(0)	3161(0)
	24(0)	321(0)	764(4)	852(0)	1222(0)	3162(0)
	37(0)	328(0)	767(41)	977(9)	1222(0)	3162(1)
	53(6)	335(1)	774(26)	983(8)	1331(24)	3163(0)
	62(0)	351(2)	777(15)	986(18)	1341(6)	3164(0)
	67(1)	374(4)	782(29)	990(8)	1344(1)	3175(2)
	71(2)	380(1)	791(4)	991(10)	1348(3)	3175(1)
	91(3)	400(2)	793(4)	995(10)	1349(1)	3175(4)
	94(18)	496(2)	798(7)	1027(4)	1352(1)	3177(2)
	117(2)	539(8)	800(1)	1029(1)	1392(4)	3178(3)
	169(2)	544(1)	801(1)	1033(0)	1399(1)	3178(2)
	171(2)	551(2)	805(1)	1034(0)	1401(4)	3188(3)
	191(0)	556(0)	809(1)	1037(0)	1408(1)	3188(3)
	199(0)	559(0)	834(0)	1040(0)	1409(3)	3188(1)
	281(3)	561(0)	839(1)	1103(7)	1413(1)	
	289(2)	749(24)	841(0)	1105(5)	1738(436)	
	293(5)	758(30)	847(0)	1106(3)	3160(0)	

Table S24. Harmonic vibrational frequencies (in cm^{-1}) and infrared intensities (in parentheses, in km/mol) for the Co1-2S structure by the BP86 method

A	32(0)	354(0)	765(8)	849(1)	1218(0)	3160(0)
	36(0)	358(0)	769(49)	849(0)	1220(0)	3161(0)
	43(0)	360(0)	774(50)	972(4)	1221(0)	3162(0)
	64(0)	379(0)	778(11)	975(6)	1340(12)	3162(0)
	67(0)	381(0)	785(3)	975(23)	1342(12)	3163(0)
	73(0)	388(0)	788(8)	993(2)	1345(2)	3172(2)
	97(0)	406(2)	789(1)	994(14)	1348(1)	3173(2)
	98(0)	410(3)	793(5)	995(14)	1350(0)	3174(4)
	132(0)	500(3)	800(2)	1027(2)	1351(0)	3176(2)
	196(1)	542(2)	800(2)	1030(2)	1389(4)	3177(4)
	197(2)	545(2)	804(1)	1032(0)	1391(4)	3178(3)
	200(0)	547(0)	805(2)	1036(0)	1393(7)	3186(3)
	202(0)	550(0)	807(2)	1036(1)	1410(0)	3187(2)
	203(0)	555(1)	834(0)	1038(1)	1412(2)	3187(1)
	324(6)	556(0)	838(1)	1101(13)	1412(1)	
	326(6)	753(11)	843(2)	1101(13)	1739(426)	
	343(14)	758(10)	847(1)	1102(1)	3158(0)	

Table S25. Harmonic vibrational frequencies (in cm^{-1}) and infrared intensities (in parentheses, in km/mol) for the Ni1-1D structure by the BP86 method

A	18(0)	288(0)	753(60)	844(0)	1219(0)	3162(0)
	29(0)	298(0)	756(56)	852(1)	1220(0)	3163(0)
	35(0)	310(0)	761(38)	973(7)	1221(0)	3163(1)
	53(0)	339(5)	764(24)	976(11)	1327(1)	3163(0)
	62(0)	357(2)	768(8)	985(10)	1332(0)	3165(0)
	65(2)	381(0)	775(19)	987(11)	1334(1)	3173(2)
	68(2)	416(10)	789(15)	992(9)	1339(16)	3175(4)
	90(0)	457(1)	798(1)	995(20)	1340(4)	3178(1)
	106(0)	502(0)	799(3)	1025(1)	1348(9)	3179(2)
	119(6)	549(1)	801(3)	1027(1)	1389(3)	3179(0)
	144(0)	550(2)	805(1)	1030(1)	1391(3)	3179(4)
	179(0)	556(0)	806(3)	1033(1)	1401(4)	3188(4)
	186(0)	558(0)	812(1)	1033(3)	1403(2)	3189(3)
	210(0)	560(1)	831(1)	1036(1)	1414(0)	3190(2)
	251(1)	565(1)	836(1)	1102(4)	1416(1)	
	271(5)	739(14)	838(0)	1102(3)	1761(438)	
	281(0)	746(5)	843(0)	1103(1)	3159(1)	

Table S26. Harmonic vibrational frequencies (in cm^{-1}) and infrared intensities (in parentheses, in km/mol) for the Ni1-2Q structure by the BP86 method

A2,-27(0)	B,266(5)	E,750(45)	E,845(0)	A2,1221(0)	E,3163(0)
E,-22(0)	E,283(0)	E,763(81)	A1,848(0)	E,1222(0)	E,3163(0)
E,-22(0)	E,283(0)	E,763(81)	E,982(1)	E,1222(0)	E,3163(0)
A,62(0)	E,338(8)	E,767(2)	E,982(1)	E,1335(8)	E,3163(0)
A,62(0)	E,338(8)	E,767(2)	A1,983(39)	E,1335(8)	A1,3164(0)
A,67(0)	A1,351(0)	A1,768(6)	A2,988(0)	A2,1337(0)	E,3176(0)
E,85(0)	E,402(10)	A1,775(11)	E,990(18)	A1,1340(0)	E,3176(0)
E,85(0)	E,402(10)	A2,803(0)	E,990(18)	E,1343(1)	A1,3177(6)
A2,108(0)	A1,477(1)	E,808(1)	E,1030(3)	E,1343(1)	A2,3177(0)
E,162(0)	A2,561(0)	E,808(1)	E,1030(3)	E,1398(1)	E,3178(3)
E,162(0)	E,561(0)	A1,810(0)	A2,1032(0)	E,1398(1)	E,3178(3)
A1,175(0)	E,561(0)	E,811(3)	A1,1037(0)	A1,1399(6)	E,3189(4)
E,187(0)	A1,566(0)	E,811(3)	E,1037(2)	A2,1406(0)	E,3189(4)
E,187(0)	E,566(1)	A2,833(0)	E,1037(2)	E,1408(2)	A1,3190(1)
B,263(0)	E,566(1)	E,839(0)	E,1104(2)	E,1408(2)	
B,263(0)	A2,746(0)	E,839(0)	E,1104(2)	A1,1748(398)	
B,265(0)	E,750(45)	E,845(0)	A1,1104(0)	A2,3162(0)	

Table S27. Harmonic vibrational frequencies (in cm^{-1}) and infrared intensities (in parentheses, in km/mol) for the Co-1T structure by the BP86 method

A	14(0)	340(1)	760(10)	961(17)	1210(0)	3151(0)
	22(0)	352(0)	768(1)	961(2)	1211(0)	3152(0)
	31(0)	383(1)	769(8)	965(14)	1313(1)	3152(1)
	36(0)	391(4)	774(6)	976(13)	1315(1)	3155(1)
	54(0)	420(1)	780(9)	976(10)	1319(0)	3162(1)
	59(1)	527(2)	780(5)	978(12)	1338(11)	3163(1)
	65(0)	532(1)	785(1)	1019(4)	1339(4)	3165(1)
	76(0)	534(0)	788(0)	1021(0)	1344(0)	3167(3)
	78(0)	541(0)	789(2)	1023(0)	1379(2)	3167(2)
	144(2)	544(1)	790(1)	1029(4)	1379(0)	3167(5)
	155(0)	552(0)	826(3)	1030(1)	1380(3)	3176(2)
	204(1)	725(5)	828(2)	1032(0)	1394(6)	3177(2)
	294(3)	729(9)	829(0)	1085(25)	1394(3)	3177(2)
	306(1)	736(9)	831(0)	1085(23)	1395(3)	
	325(6)	749(31)	833(0)	1087(9)	3145(2)	
	336(2)	752(45)	834(0)	1209(0)	3150(1)	

Table S28. Harmonic vibrational frequencies (in cm^{-1}) and infrared intensities (in parentheses, in km/mol) for the Co-2S structure by the BP86 method

A	-34(0)	355(0)	769(37)	972(0)	1216(0)	3157(1)
	-23(0)	372(2)	769(0)	974(22)	1218(0)	3160(0)
	24(0)	384(0)	775(2)	975(11)	1327(1)	3162(0)
	33(0)	410(4)	781(23)	979(18)	1338(0)	3164(1)
	54(1)	446(1)	784(0)	984(10)	1341(0)	3168(3)
	68(0)	525(0)	790(4)	988(11)	1344(3)	3169(4)
	87(0)	531(2)	791(4)	1025(3)	1348(0)	3173(1)
	90(0)	543(0)	795(3)	1026(0)	1350(3)	3173(3)
	98(1)	544(1)	802(0)	1032(1)	1383(1)	3178(2)
	164(1)	547(1)	805(2)	1034(0)	1389(2)	3183(2)
	198(1)	552(1)	822(0)	1036(0)	1392(4)	3183(1)
	223(1)	745(13)	829(0)	1036(1)	1396(2)	3189(2)
	294(1)	756(1)	835(1)	1090(16)	1402(0)	3194(2)
	304(3)	760(18)	838(0)	1096(18)	1403(4)	
	326(0)	761(32)	838(1)	1100(7)	3149(0)	
	333(8)	766(16)	841(0)	1213(0)	3153(0)	

Table S29. Harmonic vibrational frequencies (in cm^{-1}) and infrared intensities (in parentheses, in km/mol) for the Ni-1Q structure by the BP86 method

A	21(0)	307(0)	736(1)	936(7)	1205(0)	3128(1)
	31(0)	311(1)	741(28)	953(20)	1208(0)	3130(0)
	47(0)	325(0)	752(54)	962(13)	1294(0)	3136(2)
	59(0)	349(4)	755(37)	966(3)	1310(4)	3142(2)
	90(0)	368(1)	760(2)	968(11)	1312(1)	3146(2)
	96(0)	548(0)	770(0)	969(12)	1322(1)	3150(1)
	106(0)	558(0)	783(1)	1015(0)	1323(2)	3158(3)
	127(2)	566(1)	789(2)	1020(1)	1349(0)	3158(7)
	146(0)	567(0)	795(1)	1022(1)	1364(1)	3159(3)
	162(0)	576(1)	802(0)	1027(5)	1372(1)	3165(4)
	166(0)	593(2)	816(2)	1029(0)	1382(1)	3168(5)
	171(1)	693(22)	820(1)	1034(0)	1387(3)	3169(2)
	198(0)	707(2)	821(1)	1062(7)	1389(1)	3176(3)
	213(0)	715(36)	826(1)	1067(6)	1405(2)	
	222(0)	716(6)	830(1)	1083(6)	2945(2)	
	255(1)	722(14)	843(0)	1197(0)	3126(0)	

Table S30. Harmonic vibrational frequencies (in cm^{-1}) and infrared intensities (in parentheses, in km/mol) for the Ni-2D structure by the BP86 method

A	-31(0)	285(0)	754(15)	975(16)	1219(0)	3158(0)
	-19(0)	291(0)	759(31)	979(4)	1220(0)	3161(0)
	8(0)	358(3)	765(25)	982(10)	1321(2)	3164(1)
	41(0)	370(7)	776(28)	983(13)	1331(0)	3167(0)
	45(0)	413(1)	793(1)	983(4)	1331(1)	3172(3)
	58(0)	542(0)	797(0)	985(28)	1333(0)	3175(3)
	65(1)	544(2)	797(1)	1026(0)	1339(0)	3175(2)
	81(0)	545(0)	799(1)	1028(1)	1342(1)	3176(3)
	94(0)	547(1)	802(0)	1030(1)	1394(4)	3177(2)
	170(2)	555(0)	809(0)	1031(1)	1397(0)	3180(2)
	173(0)	559(0)	822(0)	1033(1)	1399(2)	3186(4)
	177(0)	739(21)	830(0)	1036(0)	1399(5)	3189(4)
	248(0)	743(32)	831(0)	1095(4)	1401(4)	3190(3)
	251(2)	745(39)	835(0)	1100(4)	1404(3)	
	271(0)	745(46)	837(0)	1101(3)	3156(0)	
	275(1)	751(8)	841(0)	1218(0)	3157(0)	

Table S31. Wiberg bond indices for the M-M bonds in $Cp_3M_3(CO)_n$ (M=Co, Ni, n=3, 2, 1, 0)

complex	M-M Wiberg bond index			M-M bond length			Bond order		
	M12	M23	M31	M12	M23	M31	M12	M23	M31
**Co3-1S**	0.32	0.30	0.41	2.44	2.44	2.48	1	1	1
**Co3-2T**	0.24	0.24	0.24	2.48	2.38	2.48	1	1	1
**Co3-3S**	0.40	0.40	0.40	2.40	2.40	2.40	1	1	1
**Co3-4T**	0.28	0.34	0.27	2.42	2.49	2.43	1	1	1
**Co2-1T**	0.32	0.33	0.41	2.34	2.36	2.41	1	1	1
**Co2-2S**	0.54	0.38	0.37	2.21	2.41	2.39	2	1	1
**Co1-2S**	0.66	0.66	0.66	2.25	2.25	2.25	2	2	2
**Co1-2S**	0.66	0.66	0.66	2.25	2.25	2.25	2	2	2
**Co-1T**	0.54	0.54	0.54	2.31	2.31	2.31	2	2	2
**Co-2S**	1.05	0.97	0.59	2.14	2.15	2.48	3	3	2
**Ni3-1D**	0.23	0.23	0.20	2.51	2.52	2.65	1	1	1
**Ni3-2D**	0.11	0.26	0.17	2.85	2.36	2.63	0	1	1
**Ni3-3Q**	0.20	0.20	0.20	2.51	2.51	2.51	1	1	1
**Ni3-4Q**	0.18	0.19	0.19	2.61	2.51	2.52	1	1	1
**Ni2-1D**	0.23	0.23	0.23	2.39	2.39	2.39	1	1	1
**Ni2-2Q**	0.21	0.21	0.25	2.45	2.43	2.31	1	1	1
**Ni1-1D**	0.38	0.38	0.39	2.29	2.29	2.29	1	1	1
**Ni1-2Q**	0.32	0.32	0.32	2.33	2.33	2.33	1	1	1
**Ni-1Q**	0.34	0.28	0.26	2.34	2.37	2.41	1	1	1
**Ni-2D**	0.51	0.51	0.57	2.36	2.36	2.32	2	2	2

Table S32. Spin densities for the M atoms in $Cp_3M_3(CO)_n$ (M = Co, Ni, n = 3, 2, 1, 0)

	**Co3-2T**	**Co3-4T**	**Co2-1T**	**Co1-1T**	**Co-1T**
Co1	1.4544	0.3770	-0.0064	-1.0288	0.6975
Co2	0.2048	0.7761	1.9113	2.0287	0.6966
Co3	0.2124	0.7864	-0.0543	0.8444	0.6974
Total	1.8716	1.9395	1.8506	1.8443	2.0915
	**Ni3-1D**	**Ni3-2D**	**Ni2-1D**	**Ni1-1D**	**Ni-2D**
Ni1	0.1667	0.5184	0.2673	0.0159	-0.0538
Ni2	0.3575	-0.0386	0.2632	0.7231	-0.0667
Ni3	0.2020	0.2076	0.2680	0.0183	0.8919
Total	0.7262	0.6874	0.7984	0.7573	0.7714
	**Ni3-3Q**	**Ni3-4Q**	**Ni2-2Q**	**Ni1-2Q**	**Ni-1Q**
Ni1	0.6883	0.6566	0.7655	0.7489	0.8875
Ni2	0.6887	0.6491	0.5555	0.7500	0.8103
Ni3	0.6889	0.7218	0.8093	0.7505	0.6632
Total	2.0659	2.0274	2.1303	2.2494	2.3610
